# Microbial communities of a variety of 75 homemade fermented vegetables

**DOI:** 10.3389/fmicb.2023.1323424

**Published:** 2023-12-15

**Authors:** Anne Thierry, Marie-Noelle Madec, Victoria Chuat, Anne-Sophie Bage, Olivier Picard, Cécile Grondin, Olivier Rué, Mahendra Mariadassou, Laurent Marché, Florence Valence

**Affiliations:** ^1^INRAE, Institut Agro, UMR STLO, Rennes, France; ^2^INRAE, Université de Montpellier, Institut Agro, URM SPO, Montpellier, France; ^3^Université Paris-Saclay, INRAE, MaIAGE, Jouy-en-Josas, France; ^4^Université Paris-Saclay, INRAE, BioinfOmics, MIGALE Bioinformatics Facility, Jouy-en-Josas, France; ^5^INRAE, UA1194, Jouy-en-Josas, France

**Keywords:** sauerkraut, lactic acid bacteria, yeast, fermented food, citizen science, spontaneous fermentation

## Abstract

Fermentation is an ancient practice of food preservation. Fermented vegetables are popular in Eastern European and Asian countries. They have received a growing interest in Western countries, where they are mainly manufactured at domestic and artisanal scales and poorly characterized. Our aim was to investigate the microbial communities and the safety of French homemade fermented vegetables, in the frame of a citizen science project. Fermented vegetables and the data associated with their manufacture were collected from citizens and characterized for pH, NaCl concentration, and microbiology by culturomics and 16S DNA metabarcoding analysis. Lactic acid bacteria (LAB) and yeast isolates were identified by 16S rRNA gene sequencing and D1/D2 domains of the large subunit of the rRNA gene, respectively. The 75 collected samples contained 23 types of vegetables, mainly cabbage, followed by carrots and beets, and many mixtures of vegetables. They were 2 weeks to 4 years old, and their median pH was 3.56, except for two samples with a pH over 4.5. LAB represented the dominant viable bacteria. LAB concentrations ranged from non-detectable values to 8.7 log colony-forming units (CFU)/g and only depended on the age of the samples, with the highest most frequently observed in the youngest samples (<100 days). The 93 LAB isolates identified belonged to 23 species, the two mains being *Lactiplantibacillus pentosus/plantarum* and *Levilactobacillus brevis*. The other microbial groups enumerated (total aerobic bacteria, halotolerant bacteria, Gram-negative bacteria, and acetic acid bacteria) generally showed lower concentrations compared to LAB concentrations. No pathogenic bacteria were detected. Viable yeasts were observed in nearly half the samples, at concentrations reaching up to 8.0 log CFU/g. The 33 yeast clones identified belonged to 16 species. Bacterial metabarcoding showed two main orders, namely, *Lactobacillales* (i.e., LAB, 79% of abundance, 177 of the 398 total ASVs) and *Enterobacterales* (19% of abundance, 191 ASVs). Fifteen LAB genera were identified, with *Lactiplantibacillus* and *Levilactobacillus* as the most abundant, with 41 and 12% of total reads, respectively. *Enterobacterales* members were mainly represented by *Enterobacteriaceae* and *Yersiniaceae*. This study is the first wide description of the microbiota of a large variety of homemade fermented vegetables and documents their safety.

## 1 Introduction

Fermentation is an ancestral process of food preservation used all over the world. It makes food products available throughout the year by extending their shelf-life despite the seasonal production of most raw materials, and, for vegetables, preserves a source of vitamins when fresh vegetables are not available. Fermentation of vegetables has recently received a renewed interest because of their natural and health-promoting image, the low cost of the process, and its potential for innovation (Thierry et al., 2023). A very large variety of vegetables can be fermented (Gänzle, 2022). Fermented vegetables are widely consumed in the world with emblematic products such as kimchi and many other traditional fermented vegetables in Asia, sauerkraut and various pickles in Eastern and Central Europe, and olives in Southern Europe. However, apart from sauerkraut and olives, fermented vegetables are little consumed in Western Europe (Sõukand et al., 2015; Medina-Pradas et al., 2017; Tamang et al., 2020).

Vegetables are generally spontaneously fermented by the microorganisms naturally present in the raw materials (Di Cagno et al., 2013), but practices vary according to the country. In Korea, the majority of consumed kimchi is homemade and results from spontaneous fermentation (Song et al., 2020), while the Chinese *suancai*, a traditional fermented product made from different vegetables, has gradually evolved from a spontaneous fermentation to an inoculated one (He et al., 2021). Some manufacturers use selected starters, but these practices remain anecdotal even for sauerkraut production at a large scale (Medina-Pradas et al., 2017). Vegetable fermentation is also called ‘lactofermentation’ because it relies on the growth of lactic acid bacteria (LAB), which ferment vegetable carbohydrates into lactic acid as their main end-product, thus inhibiting most other bacterial groups. LAB are widespread in nature and are associated with nutrient-rich habitats such as plants, soil, animals, and humans (Holzapfel and Wood, 2014). They, however, represent less than 1% of the total bacterial population of raw vegetables, whose microbiota is generally dominated by aerobic bacteria such as pseudomonads, enterobacteria, and yeasts (Buckenhueskes, 2015). The microbiota of vegetables significantly changes during the course of fermentation, in response to the changing environmental conditions within the fermenting substrate. In the case of sauerkraut, for example, several stages have been described, with a gradual decrease of strictly aerobic and facultatively anaerobic bacteria such as *Enterobacteriaceae*, accompanied by a gradual increase of LAB. LAB grow in succession, *Leuconostoc mesenteroides* typically initiates lactic fermentation, and homofermentative lactobacilli succeed leuconostoc, followed in extended fermentations by the growth of heterofermentative LAB species such as *Levilactobacillus brevis* (Buckenhueskes, 2015). Similarly, in fermented carrot juice, *Enterobacteriaceae* were shown to be among the first families to develop before being rapidly outcompeted by LAB between 3 and 13 days of fermentation (Wuyts et al., 2018). At the stage of consumption, LAB are the dominant bacterial microbiota, represented by *Leuconostoc* spp., *Lactobacillus*-related, *Weissella*, *Enterococcus*, and *Pediococcus* genera, with the most frequently isolated species *Lactiplantibacillus plantarum*, a ubiquitous and metabolically versatile species (Ashaolu and Reale, 2020; Torres et al., 2020). The presence of other microorganisms has also been reported in fermented vegetables, such as yeasts (Ballester et al., 2022) and *Archaea* (Park et al., 2009). If moderately explored in the past, the microbial diversity of fermented vegetables, such as other fermented foods, has largely benefited from recent advances in high-throughput sequencing technology (Cocolin and Ercolini, 2008; De Filippis et al., 2018). Sauerkraut and kimchi are the most studied ones (Jung et al., 2014; Lee et al., 2017; Zabat et al., 2018). The combination of culture-dependent and independent approaches is increasingly used as it brings a comprehensive view of fermented vegetable microbiota, as done for example for kimchi (Lee et al., 2017), in which the main LAB responsible for fermentation are members of the *Weissella*, *Lactobacillus*, and *Leuconostoc* genera (Song et al., 2020).

Homemade fermented vegetables are manufactured using a quite simple process, although some key points have to be respected. Vegetables are diced or shredded, more or less finely, and salted, by adding either brine (at ~30 g/L NaCl) or dried salt (at approx. 10 to 20 g NaCl/kg vegetables), to reach a final salt concentration of around 1 to 2% of the total mix. The main function of salting is to withdraw water and nutrients from vegetable tissue, which also provides microorganisms with the substrates they need for growth. Salt can be added as dry salt or as brine, depending on the capacity of vegetables to release enough juice to recover vegetables (Thierry et al., 2023). Sliced vegetables are filled and pressed in glass jars to eliminate air pockets, thus promoting an anaerobic environment. Products are let to ferment at ambient temperature for at least 3 to 4 weeks before being consumed or further stored at lower temperatures. A huge number of recipes are available in cookbooks, e.g., Katz (2012), and on the web in dedicated blogs. Recipes suggest the use of various vegetable mixes, spices, and condiments, but they all rely on this basic process. In Western Europe if sauerkraut is mostly produced at an industrial or semi-industrial scale, the renewed interest in fermented vegetables has been accompanied by an increase in homemade fermented vegetables, which have been poorly characterized, to the best of our knowledge.

The aim of the present study was to investigate the microbiota and microbial safety of French homemade fermented vegetables. To collect a large number of samples from citizens who produce fermented vegetables for their personal consumption, a crowdsourcing approach was used, in the frame of the citizen science project FLEGME (for “Fermentation des LEGuMEs,” French for “vegetable fermentation”). A total of 75 various homemade fermented vegetables were analyzed by combining targeted culturomics and a culture-independent approach to characterize their microbiota. The presence of pathogenic bacteria was also investigated, and collections of LAB and yeast isolates representative of the fermented vegetable microbiota were constituted.

## 2 Materials and methods

### 2.1 Collection of the samples and associated data

A total of 75 homemade fermented vegetables were collected from February to October 2020. Samples were collected from French citizens who manufacture fermented vegetables for their personal consumption. Citizens were let free to choose the sample they wish to send into analysis. Half of the samples were sent by mail either in the Le Parfait-type glass jars in which they were prepared, and the other half after being sampled in a 250 mL sterile plastic bottle/vial provided to the citizens, along with sampling instructions.

In parallel, citizens were asked to complete an online survey containing 64 questions about the raw materials used, the manufacturing and fermentation practices, and the recipe used. These data were used to generate 40 qualitative and 17 quantitative variables, either directly extracted or derived from the data collected from the survey. Concerning qualitative variables, for example, the vegetables used were classified into four categories according to the plant organ (root/bulb, leaf/stem, fruit, or mixed), the cutting of the vegetables was classified into two types (rough or thin, see further details below). As for quantitative variables, we calculated the age of the sample, i.e., the time between the manufacture and the analysis of samples. We also calculated an expected NaCl concentration from the recipe supplied by each citizen, either directly in the case of dry salting or by multiplying the concentration of NaCl in brine by a factor of 0.3, which considers that the ratio brine:vegetable is approximately 1:2. All the details collected and variables created are compiled in Supplementary Table S1.

Samples were stored at 4°C upon receipt, for a period of 1 to 6 days before analysis. Then, they were subjected to pH measurements and microbial enumerations and frozen at −20°C as separate samples until other analyses (DNA extraction for metabarcoding analysis and biochemical analyses) were performed.

### 2.2 Biochemical and microbiological characterization

The pH of the samples was measured with a pH meter (Hanna Instruments HI 2020-02).

The NaCl concentrations were determined from sodium content measured using an inductively coupled plasma-optical emission spectrometer (ICP-OES) (iCAP 7200, Thermo Fisher Scientific, Courtaboeuf, France), as previously described (Martin et al., 2022). Before analysis, juice samples were centrifugated at 18,000 *g* for 10 min at 4°C, and the supernatants were 1000-fold diluted in a 2% v/v HNO_3_ (Thermo Fisher Scientific, Waltham, MA, USA).

For microbial analyses by plate counting, samples of 10 g of fermented vegetables (5 g of juice plus 5 g of drained vegetables) were suspended in preheated (48°C) 90 mL of a Tryptone Salt diluent (TS; sodium chloride 8.5 g/L, tryptone 1 g/L) and homogenized in a filter bag (BagPage+, Interscience), in which vegetable debris was separated from the filtrate. The filtrate was then serially diluted in sterile TS diluent. 100 μL of the serial dilution was plated on eleven different nutritive and selective media and incubated under aerobic (air atmosphere) or anaerobic conditions (Anaerocult^®^ A, Merck, Darmstadt, Germany) at 37°C, 30°C, or 25°C, depending of the medium, according to Supplementary Table S2.

In addition, four pathogens, namely, *Escherichia coli*, coagulase-positive staphylococci (*Staphylococcus aureus*), *Salmonella*, and *Listeria monocytogenes*, were searched by a subcontracted laboratory (LABOCEA, Fougères, France), following the ISO 16649-2, ISO 6888-2, BRD 07/11-12/05, and AES 10/03-09/00 standards, respectively.

*Bacillus cereus* typical colonies on BCA were further examined, by observing their aspect on the agar medium Compass Bacillus cereus (Biokar), incubated at 30°C for 24 h and 48 h, microscopical observation, and *panC* gene sequencing.

For a few samples positive on TSN and/or for which high concentrations of spore-forming bacteria were observed on BHI-YE, spore-forming bacteria were also enumerated after a 10 min-heat treatment at 80°C on BCP containing, per liter, peptone 5 g, beef meat extract 3 g, lactose 10 g, bromocresol purple 0.025 g, and agar 13 g (Biokar Diagnostics, France) and meat-liver glucose agar (VF, Biokar) according to the NF V08-602 standard, both incubated for 48 h at 37°C.

### 2.3 Microbial isolation and identification by sequencing

To collect LAB strains, 1 to 3 isolates were picked up from MRS plates containing 20 to 100 colonies, according to the visual aspect of the colonies (size, color, morphology), to favor the diversity of the isolates collected. Yeast isolates were collected from OGAc plates following the same methodology. Bacteria and yeast were identified by 16S rRNA gene and D1/D2 domain of 26S rRNA gene sequencing, respectively.

For bacterial identification, the 16S rRNA gene was amplified by using W001 and W002 primers according to Godon et al. (1997), using TM thermocycler C1000 (Bio-Rad, Australia). Amplified PCR products were purified and sequenced by the Sanger method (LGC Genomics GmbH, Berlin). Sequences were assembled using Geneious software, and contigs were blasted on NCBI. For yeast identification, the D1/D2 LSU rRNA gene and ITS, which included ITS1–5.8S–ITS2, were amplified by NL1 and NL4 (O’Donnell, 1993) and ITS1 and ITS4 (White et al., 1990), respectively, using a 2720 thermal cycler (Applied Biosystems). For strains of the *Debaryomycete* genus, part of the gene coding for actin (ACT1) was amplified by CA14-DEHA and CA5R-DEHA (Jacques et al., 2015). The resulting amplicons were sequenced on both strands by Eurofins MWG Operon (Eurofins Genomics). Sequences were assembled with the phred/phrap/consed package and compared to sequences in databases, such as GenBank and YeastIP, using the BLAST program (Amoikon et al., 2018).

### 2.4 16S DNA metabarcoding analysis

#### 2.4.1 Mock community

A mock community, i.e., a synthetic community composed of known proportions of a set of bacterial species commonly found in fermented vegetables, was designed by combining the 10 following strains: *Pediococcus pentosaceus* CIRM-BIA2073, *Levilactobacillus brevis* CIRM-BIA2321, *Lactiplantibacillus plantarum* CIRM-BIA2367, *Leuconostoc mesenteroides* CIRM-BIA2378, *Lactococcus lactis* CIRM-BIA2287, *Enterococcus faecium* CIRM-BIA1821, *Enterobacter cloacae* CIRM-BIA2529, *Gluconobacter cerinus* CIRM-BIA2206, *Acetobacter persici* CIRM-BIA2399, and *Halomonas casei* CIRM-BIA2432, obtained from the CIRM-BIA collection (International Centre for Microbial Resources dedicated to Bacteria of Food Interest, INRAE, Rennes, France).^1^ The optical density (OD) of cultures was measured at 600 nm. The exact volume to collect was calculated for each strain from previously established colony-forming units (CFU) versus OD curves, to obtain a concentration of 10^7^ CFU/mL of each strain. The *ad hoc* volume of each culture was transferred to a Falcon tube and then centrifuged, and the pellet was washed and stored at −20°C until DNA extraction and sequencing.

#### 2.4.2 DNA extraction and sequencing

For fermented vegetables, DNA was extracted from the samples (*n* = 75), in duplicate, using the Nucleospin Tissue kit (Macherey-Nagel, Düren, Germany). A 10 mL aliquot of the first dilution used for plate counting was centrifuged at 12,000 *g* for 15 min at 4°C. The supernatants were discarded, and the pellets were stored at −20°C until extraction. DNA was extracted according to a protocol that includes several enzymatical and one mechanical steps, with slight modifications (Penland et al., 2021). The defrosted pellets were first resuspended in 400 μL of lysis buffer (Tris: 2.42 g/L; EDTA: 0.58 g/L; Triton X100: 10 g/L), supplemented with lysozyme (20 mg/mL, ref. L6876, Sigma-Aldrich), mutanolysin (50 U/mL, ref. M9901, Sigma-Aldrich), RNase A (0.225 mg/mL, Qiagen), and lyticase (200 U/mL, ref: SAE0098, Sigma-Aldrich). Suspensions were lysed at 37°C for 2 h. Then, the lysate was transferred to a bead tube that contained 300 mg of 0.1 mm diameter zirconium/silica beads and homogenized for 2 × 1.5 min using a Precellys Evolution homogenizer (Bertin technologies, Germany), with a cooling step on ice of 30 s between the two homogenizations. Finally, proteinase K, at a final concentration of 20 mg/mL (provided in the Nucleospin Tissue kit), was added, and the samples were further incubated at 56°C for 2 h. Then, 200 μL of B3 buffer of the Nucleospin Tissue Kit was added, and a last lysis step was performed for 10 min at 70°C. Samples were centrifugated at 6,000 *g* for 10 min, and the supernatant was collected in a new tube. The next steps were proceeded according to the manufacturer’s instructions until the DNA elution. For the mock community, the pellets were prepared and extracted as described above for vegetables After extraction, the eluted DNA was quantified in each tube using a Qubit 4 Fluorometer TM (Invitrogen, Thermo Fisher Scientific), and the ratio A260/A/280 was evaluated using a NanoDrop ND-1000 spectrophotometer (Labtech, Palaiseau, France). A control was also performed in duplicate to evaluate the contaminants in DNA extraction kits and reagents, so-called “kitomes,” with defrost pellet replaced by ultrapure water. DNA sequences were amplified in the 16S rRNA V5-V7 region for bacteria using primers 799F/1193R (Forward- AACMGGATTAGATACCCKG, Reverse-ACGTCATCCCCACCTTCC) and PCR conditions as previously described (Beckers et al., 2016). The 16S rRNA amplicons were sequenced at the Genome Quebec sequencing platform (Montreal, Quebec) using Illumina MiSeq PE250 technology, which generated 2 × 250 bp reads and a total of 2.45 Gb of data for amplicons.

#### 2.4.3 Bioinformatic analyses

The raw sequences were processed using DADA2 package v 1.20.0 (Callahan et al., 2016), following the author’s guidelines: We applied successively the functions filterAndTrim, learnErrors, dada, mergePairs, and makeSequenceTable. The resulting amplicon sequence variants (ASVs) were processed using FROGS v3.2.3 (Escudié et al., 2018; Bernard et al., 2021) to remove chimera, filter out low-abundance ASVs (< 0.005% of the total abundance), and affiliate the filtered ASVs with SILVA v138.1 as the reference databank (Quast et al., 2013). ASVs affiliated with chloroplasts or mitochondrial sequences were excluded. Finally, ASVs with multiple equally good but incompatible affiliations were flagged for inspection and manually curated using an interactive interface^2^ to either select one of the competing affiliations or conserve the ambiguity as a “multi-affiliation” tag. The ASV counts of the extraction duplicates were merged, by summing them, after checking that the duplicated clustered together, to achieve a higher total read count for the sample. Finally, 24 samples with a total read count lower than 5,000 were filtered out.

### 2.5 Statistical analyses

One-way ANOVA was performed to determine whether the microbial variables and pH differed according to the nature of the vegetable or the category of vegetable (root/bulb, leaf/stem, fruit, or mix), by using the R function *aov*. The means were then compared in a pairwise fashion using the Tukey HSD post-hoc test from the R package *emmeans.*

The chi-square tests of independence were performed to investigate (i) the relationships between qualitative variables, e.g., between the type of vegetable and the type of salting applied, and (ii) the non-linear relationships between some variables, e.g., the viable LAB numbers and the age of the samples, or the quantity of extracted DNA and the age of the samples. For this analysis, the quantitative microbial variables were binarized as follows: concentrations on MRS using [0–6] and [6–10] logCFU/g ranges and the sample age using [0–100] and [100–1600] ranges.

A principal component analysis (PCA) was performed by using the PCA function of the FactoMineR R package to illustrate the global biochemical and microbiological composition of the 75 fermented vegetables and the relationships between the different variables. A hierarchical clustering was performed on PCA data. Microbial variables with a high (>60%) proportion of null values (i.e., plate counts on YPMnp, KF, VRBG, BCA, and TSN media) were converted into qualitative variables (absence/presence). The association of qualitative variables with PCA first dimensions was investigated by using v-tests and was considered significant for v-test values of <−2 or > 2.

For 47 of the 75 samples that were correctly analyzed using metabarcoding, a multiple factor analysis (MFA) was performed by using the MFA function of the FactoMineR R package, to investigate the relationships between the variables that described the samples and those resulting from biochemical analyses (pH, NaCl concentration) or microbial analyses (enumeration data and proportion of the main taxa from metabarcoding analysis). A hierarchical clustering was performed on MFA data.

For metabarcoding data, diversity analyses were performed using the phyloseq (v1.44) R package. Data were rarefied before computing alpha diversity (observed richness, Shannon index) and beta diversity (Jaccard distance). The relationship between variables and alpha diversity was assessed using one-way ANOVA and the relationship between variables and beta diversity using permutational ANOVA (PERMANOVA, with 9999 permutations for computing *p*-values).

A non-supervised analysis was performed to explore the structures of ASV data. The counts were filtered to keep only dominant taxa (prevalence >20%, abundance >0.1% in at least one sample). A PLN-mixture (Chiquet et al., 2021) model was then fitted to the reduced dataset to cluster samples into two to six clusters. The optimal number of clusters was chosen using the ICL criteria, following the authors’ guidelines.

## 3 Results

### 3.1 A large diversity of fermented vegetables collected

The main characteristics of the 75 samples collected are given in Supplementary Table S1. Their age, i.e., the period between the day of manufacture and the day of analysis, spreads out between 2 weeks and 4 years, with a median value of 6 months.

**Figure 1:**
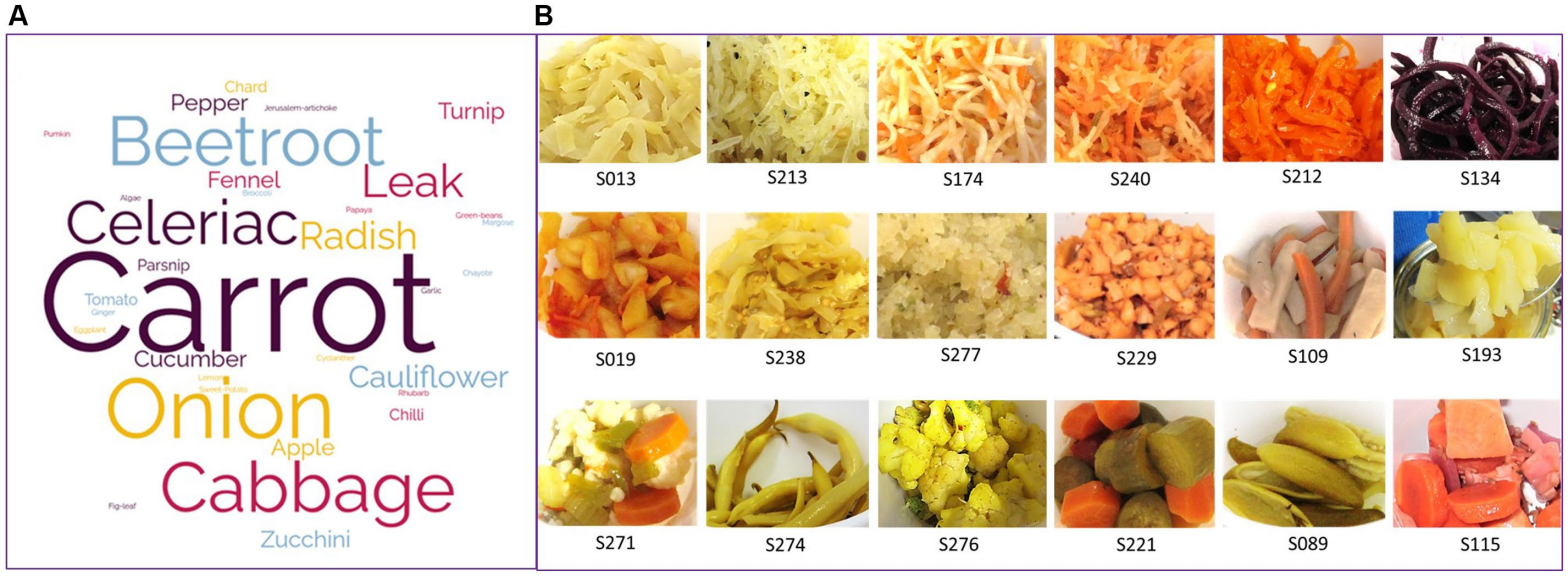
Vegetables used to manufacture the homemade fermented vegetables characterized in this study. **(A)** Word cloud scheme built from the list of all vegetables used in the 75 recipes associated with the samples received. **(B)** Pictures illustrating the different sizes of slicing, either thinly cut (grated, shredded, two upper lines) or roughly cut (into slices, dices, or simply cut in two lengthwise, bottom line).

A large diversity of fermented vegetables was collected. In total, 23 types of vegetables were used in the recipes, either pure (in 60% of the collected samples) or mixed with two to six vegetables (in 40% of samples). The most frequently used vegetables were carrots, cabbage (seven varieties), onion, beet, and celeriac, present in 36, 27, 15, 12, and 11% of samples, respectively (Figure 1A). Among the 45 samples in which pure vegetables were used, the main vegetables were cabbage (*n* = 10), carrot (*n* = 8), and beetroot (*n* = 6), but 15 other vegetables were also fermented alone. Vegetables were equally distributed between those derived from the aerial parts of vegetables (leaves, stems, fruits, 53%) and the underground ones (root, bulbs, 47%). The vegetables used came from local producers (43%), were bought in other stores (36%), or produced in private vegetable gardens (21%). They were mainly produced using organic agriculture techniques or permaculture (81%) and mainly locally [originated from local producers or vegetable gardens (21%)] (Supplementary Table S1).

The size of slicing varied according to the recipes, 61% were thinly cut (grated, shredded) and 39% were more roughly cut (into slices, dices, or simply cut in two lengthwise, for example), or let entire in the case of some small size vegetables, as illustrated by the pictures on Figure 1B.

Most (16/20) samples of cabbage and cabbage mix and all beet samples were thinly cut, while for carrots and other vegetables, about half of the samples were thinly cut, e.g., grated, and half were roughly cut, e.g., sliced, diced, or cut into stripes or chunks (Supplementary Figure S1A).

All the collected samples were salted. Coarse salt was used in most cases, with gray coarse sea salt the most frequently used. Salt was added either in brine (40 of 75 samples, with brine prepared at 10 to 30 g/L) or added as dried salt at concentrations ranging from 0 to 30 g/kg vegetables. Dry salting was markedly more frequent in cabbage and cabbage mix samples than for other vegetables (Supplementary Figure S1B). Moreover, the type of salting was also associated with the type of cutting. As expected, dry salting was more frequently applied to thinly cut vegetables (63%) than to roughly cut ones (21%) (*p* < 0.001, data not shown).

In addition, two-thirds of the recipes used to prepare the fermented vegetables included other minor ingredients, such as ginger root (27% of samples) and garlic (25%), and several herbs (bay leaf, thyme, sage, tarragon, oregano, and also, less frequently, lovage, parsley, chive, etc.) and/or spices (coriander seeds, cumin seeds, whole or ground pepper, mustard seeds, juniper berries, turmeric, caraway seeds, paprika, chili, etc.), generally used in combination (Supplementary Table S1). Other types of ingredients were also included in some of the recipes, such as soy sauce, nuoc-mam, glutinous rice flour, and algae. Up to 10 total number of ingredients (vegetables, herbs, spices, others) were used per product, with an average number of three ingredients.

Most products (71 out of 75) were prepared by spontaneous fermentation. Three were inoculated by backslopping, i.e., by adding a portion of the juice of a previous production as an inoculant, and the last one by kefir addition (Supplementary Table S1).

Six of the manufacturing practices that may influence the initial microbial community were also compiled (Supplementary Table S1). The most common were to peel vegetables (*n* = 42), to wash hands before manufacture (*n* = 34), to wash vegetables (*n* = 54), to mix the ingredients by hands (*n* = 60), to fill and compact the ingredients by hands (*n* = 52), and to not use any starters, as stated above (*n* = 71). From these factors, a “diversity score” was calculated by summing the number of factors that may influence the initial microbial community by favoring diversity and/or risks. For example, three samples (S092, S182, and S237) received the maximal score of 6 because they were prepared with no peeling, no hand washing, ingredient manipulations by hands, and no starters added, while two samples (S229 and S247) received the score of 1 because they were prepared without starters but with stricter washing and manipulating precautions. The median score was 4.

### 3.2 Biochemical characteristics of the fermented vegetables collected

**Figure 2:**
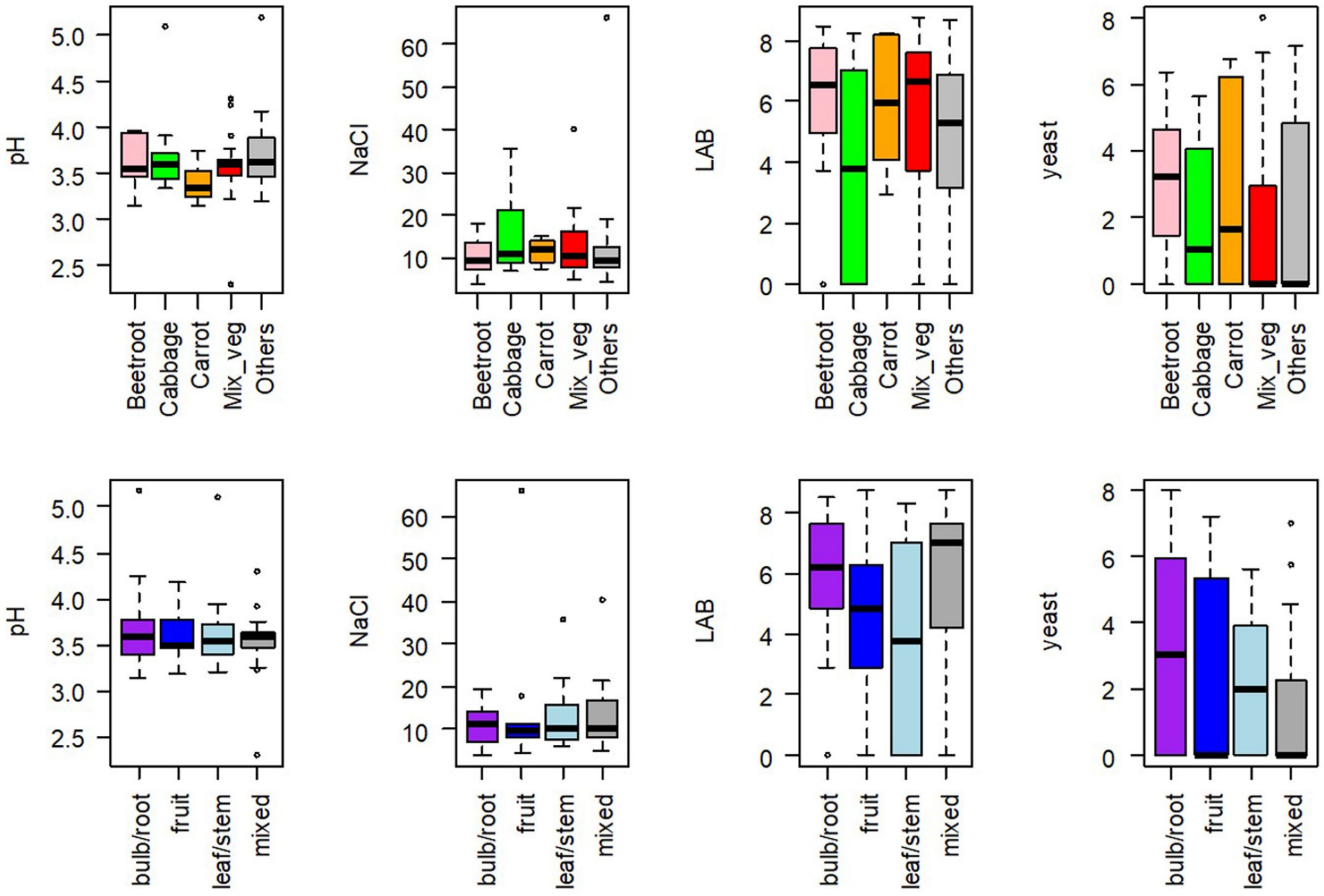
Boxplots of pH values, NaCl concentrations expressed in g/L, lactic acid bacteria (LAB) and yeast concentrations, expressed in log CFU/g, for 75 homemade fermented vegetables, according to (upper figures) the type of vegetable, used either alone: beetroot (*n* = 7), cabbage (different types, *n* = 10), carrot (*n* = 8), others (*n* = 21), or used in mixture: Mix_veg (*n* = 29); (bottom figures) the category of vegetable: bulb/root (*n* = 26), leaf/stem (*n* = 13), or fruit (*n* = 13), or mixed (*n* = 23).

The median pH value of samples was 3.56, and 73 out of 75 samples ranged between 3.14 and 4.30. The two other samples were a 39-day mixed cabbage (S276) and a 74-day turnip (S277), with a pH of 5.10 and 5.18, respectively. The pH value significantly depended neither on the type of vegetable used nor on the category of vegetable (root/bulb, leaf/stem, fruit, or mixed) (Figure 2).

NaCl concentration was calculated from Na measurements. Its median value was 10.2 g/kg juice, but it ranged between 4.0 and 66.1 g/kg juice. Only three samples contained more than 35 g/kg (S213, S237, and S238). NaCl concentration significantly depended neither on the type nor on the category of vegetable (Figure 2). We observed large discrepancies between the NaCl concentrations actually measured (NaCl) and the concentrations expected according to the calculations from the recipes collected for each sample (Declared_NaCl). The regression equation was NaCl = 0.91 × Declared_NaCl +4.16, with *R*^2^ = 0.3986. This global equation hides huge variations since (measured) NaCl values were up to 7.7-fold lower and 3.6-fold higher compared to the Declared_NaCl.

### 3.3 Composition in viable microorganisms

LAB represented the dominant population in all samples. Their median concentration was 5.87 log CFU/g but varied from non-detectable values (in 12 samples) to 8.75 log CFU/g. LAB concentrations did not significantly depend on the type or the category of vegetable (Figure 2). Moreover, no direct correlation was observed between LAB concentrations and the age of the samples, which ranged from 2 weeks to 4 years (Supplementary Figure S2). However, high LAB concentrations were significantly (*p* < 0.001, chi-square test) most frequently observed in the youngest samples. More specifically, LAB concentrations >6 log CFU/g represented 88 and 29% of samples under and over 100 days old, respectively. Enterococci, enumerated on the selective KF medium, were detected, at concentrations <5 log CFU/g, in only eight samples that all contained high LAB concentrations, and thus represented an extremely small fraction of LAB (approx. 0.001%).

The other microbial groups enumerated most generally showed lower concentrations compared to LAB concentrations (Supplementary Table S1 and Supplementary Figure S3). Total aerobic bacteria, estimated on BHI-YEn, a non-selective, rich medium that contained natamycin to prevent yeast growth, showed concentrations well correlated with concentrations on MRS, with only 9 out of 75 samples for which BHI-YEn counts exceeded MRS concentrations. Similarly, concentrations on TSANaCl, a medium that targets halotolerant bacteria, were in most cases slightly lower but of the same order of magnitude, compared to MRS concentrations, suggesting they could correspond to a halotolerant LAB fraction. Aerobic Gram-negative bacteria, counted on BHI-YEnp, were detected in two-thirds of samples, at concentrations ranging from 2.3 to 8.8 log CFU/g. They were poorly correlated and never exceeded LAB concentrations. Colonies on YPMApn, used to target acetic acid bacteria, were observed for 30 of the 75 samples, at concentrations lower or similar to LAB concentrations.

Yeasts were detected in nearly half the samples, at concentrations ranging from 2 to nearly 8 log CFU/g. Yeast concentrations did not significantly depend on the type or the category of vegetable (Figure 2).

Bile-resistant *Enterobacteriaceae*, enumerated on VRBG, were detected in only four samples. Two of them contained high bacteria concentrations on VRBG, were rather young, and had a higher pH compared to the other samples (S275, 51-day tomato, pH 4.18, and S276, a 39-day cabbage, pH 5.10). In contrast, the two other samples were quite aged and acidified samples (S135, an onion-fennel mixture aged 141 days, with a pH of 3.60; and S094, a leak-radish-green onion mixture aged more than 2 years, with a pH of 3.64).

No pathogenic bacteria were detected (*Escherichia coli*, *Salmonella*, *Listeria monocytogenes*). Coagulase-positive staphylococci were lower than 10 CFU/g in most samples (68/75), < 100 in 9 samples, and were enumerated at 480 CFU/g in only one sample (S212, grated carrots aged 56 days, with a pH of 3.74).

Aerobic total spore-forming bacteria, enumerated on BHI-YE-70, were detected in 40 of 75 samples, at concentrations ranging from 1 to 4.2 log CFU/g. The concentrations on BHI-YE-70 were of the same order of magnitude compared to that observed on BCP, another rich medium, regardless of the heat treatment applied (70°C for 15 min or 80°C for 10 min), tested for comparison for a few samples (data not shown). Thirteen samples were positive on BCA agar, of which three exhibited one or two typical colonies (S071, S089, and S185). These clones were identified as members of the *Bacillus cereus* groups II and IV, which may include cytotoxic strains. Five samples showed 1 to 2 log CFU/g on TSN. Only one typical black colony was observed on this medium (sample S273, colony not further identified). The highest concentrations of spore-forming bacteria were observed for a leak/radish /onion sample, S094. For this sample, colonies were observed on several media that targeted spore-forming bacteria, including VF which targets the anaerobic sulfite-reducing bacteria. The concentrations for the S094 sample on BHI-YE, BCP, BCA, and VF were 5.2, 4.0, 2.6, and 4.5 log CFU/g, respectively, but no colonies were observed on TSN used to enumerate clostridia.

### 3.4 Multivariate analysis based on biochemical and enumeration results for all samples

**Figure 3:**
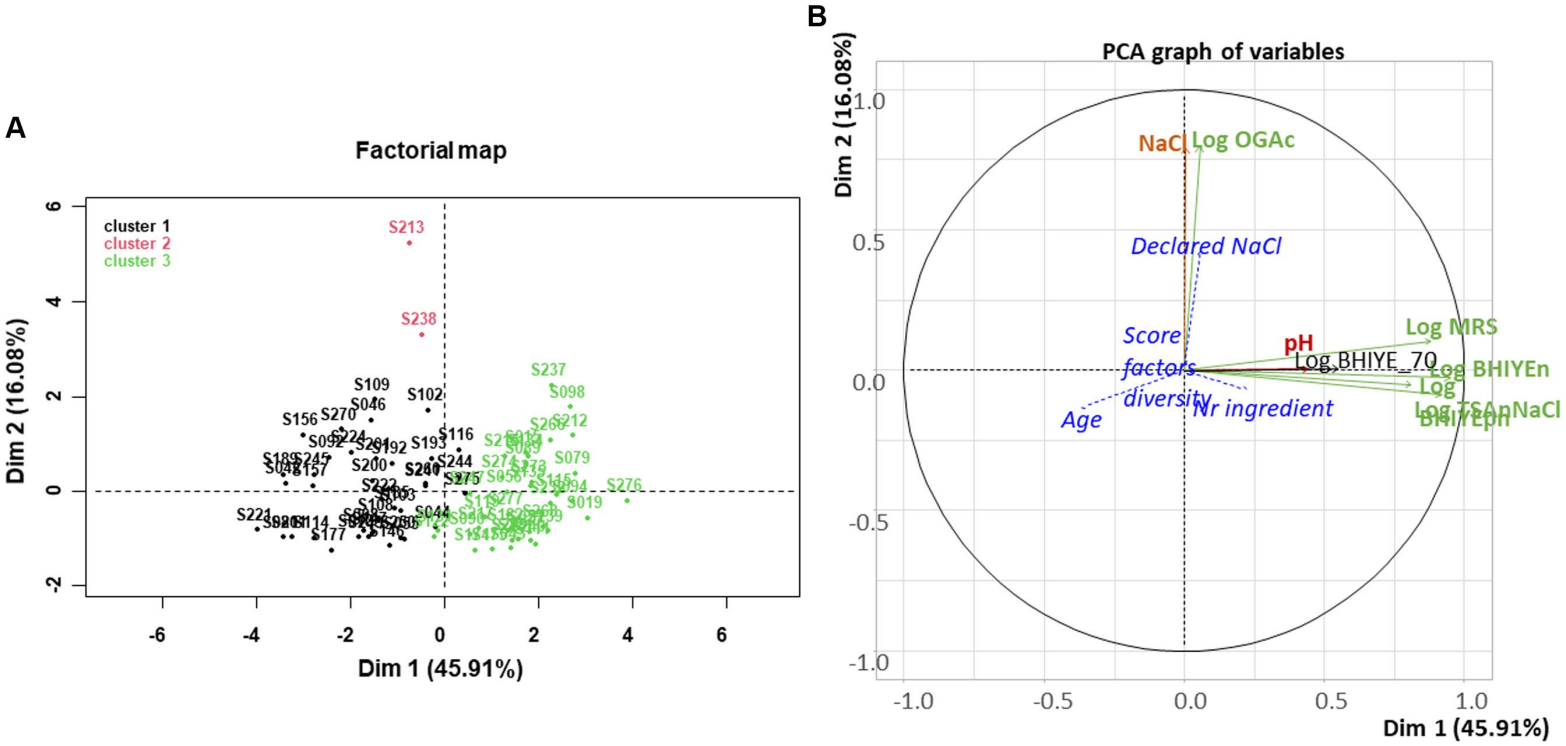
Principal component analysis performed for the 75 homemade or artisanal fermented vegetable samples collected. **(A)** Factorial map: individuals are colored according to a hierarchical clustering made on PCA data. **(B)** Graph of variables: active variables are microbial plate counts enumerated on different media (see Supplementary Table S2), shown in green, and in black for enumeration after a 70°C and 15 min treatment, and pH and measured NaCl concentration, shown in red. Four variables that describe samples are projected as supplementary variables, shown in blue italics and blue dotted lines: the age of the samples, their expected NaCl content calculated from declaration in the corresponding recipes, the number of ingredients in the recipes, and the “diversity score” corresponding to the number of practices and factors that may influence the initial microbial community (see text).

A PCA was done to investigate the structure of the dataset, and the possible relationships between the variables that describe sample composition, used as active variables, and some qualitative and quantitative variables that described their manufacture (age, type of vegetable, type of salting, type of cutting, etc.), used as supplementary variables (Figure 3). The first axis, which explained 45.9% of total variability, separates the samples based on their concentrations of alive bacteria, including LAB, on the four main media used. Bacterial alive concentrations were positively associated with axis 1 (*r* = +0.95, +0.92, +0.88, and + 0.81 for total aerotolerant bacteria enumerated on BHI-YEn, halotolerant bacteria, LAB, and Gram-negative bacteria, respectively). The first axis was also weakly negatively associated with the age of the samples (*r* = −0.37). The second axis (16.1% of total variability) was positively associated with yeast concentrations and NaCl content (*r* = +0.80 and + 0.79, respectively). PCA also highlights the poor correlation between the analyzed and declared NaCl concentrations as both variables were not close on the PCA variable map. The three other quantitative variables projected were poorly represented, indicating that the age of the samples, the number of ingredients, and the “diversity score” were not correlated with the microbial and biochemical composition of samples. Concerning the other microbial groups targeted, used as qualitative supplementary variables, the first dimension was positively associated with the presence of colonies on VRBG, KF, and TSN (v-test of 2.37, 3.87, and 2.72, respectively), suggesting that the presence of these minor bacterial groups was related to that of the main bacterial groups.

Three clusters were distinguished by a hierarchical clustering performed on PCA data (Figure 3). The first main cluster (n°1 in black on the left hand) contained samples with low bacterial concentrations and was rather aged. The second cluster (in red on the top of the PCA map) contained only two very salty samples (S213 and S238, 6.6 and 4.0% NaCl, respectively), which both also contained rather high yeast concentrations. It is important to underline, however, that NaCl concentration and yeast concentrations were globally not correlated. The second main cluster (n°3 shown in green on the right hand) contained samples with high bacterial concentrations and a rather low age, in general.

The type of salting appeared associated with the second PCA dimension, with dry salting significantly (v-test = 2.2) positively associated with the second dimension. None of the other qualitative variables appeared significantly represented in any of the first dimensions, including the type of vegetable, the category of vegetable (bulb/root, leaf/stem, fruit, or mixed), and the type of cutting (thin or rough).

### 3.5 Identification of LAB and yeast isolates

**Figure 4:**
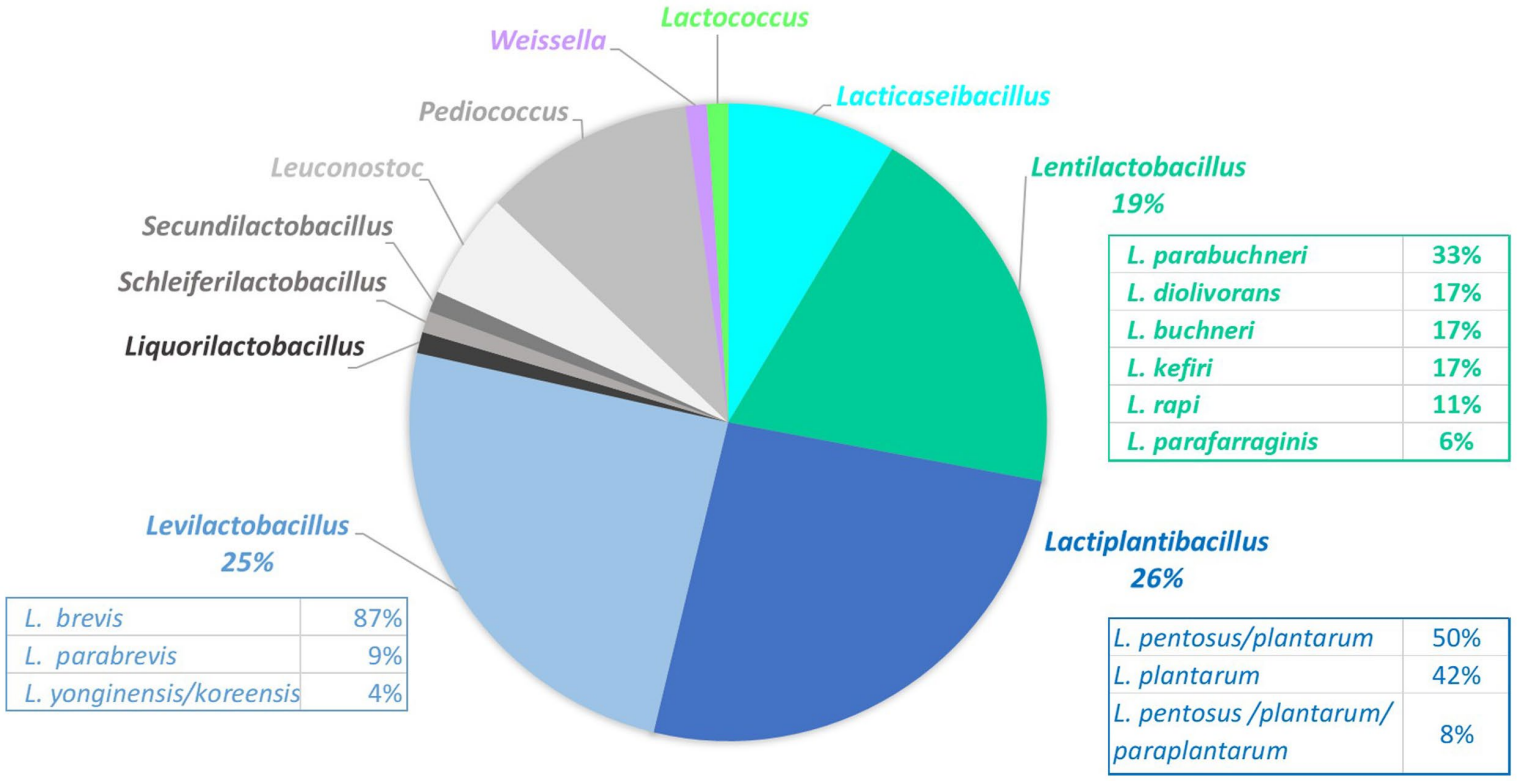
Proportions of the 93 lactic acid bacteria isolates collected on the MRS plates from 59 fermented vegetable samples and identified by 16S rRNA gene sequencing.

A total of 93 LAB clones isolated from MRS plates were collected from 59 samples and identified (Supplementary Table S3). In total, 11 genera were identified, represented by 23 taxa (Figure 4). The three most abundant genera were *Lactiplantibacillus* (26% of total isolates, represented by *L. plantarum/paraplantarum/pentosus* isolates), *Levilactobacillus* [25% of total isolates with mainly *L. brevis* isolates (87%)], and *Lentilactobacillus* (19%, represented by *L. parabuchneri/buchneri* and *L. diolivorans*) (Figure 4). The following genera were *Pediococcus* (11%, mainly *P. parvulus*), *Lacticaseibacillus* (9%, *L. paracasei/casei*), and *Leuconostoc* (5%, represented by *L. mesenteroides/pseudomesenteroides*). One unique isolate was also identified for each of the five species *Secundilactobacillus malefermentans, Lactococcus lactis, Liquorilactobacillus satsumensis, Schleiferilactobacillus harbinensis*, and *Weissella viridescens*.

Concerning yeasts, 33 clones were isolated from 17 samples, representing a high diversity with 12 genera and 16 species (Supplementary Table S4). The three prevalent genera were *Kazachstania*, represented by the three species *K. barnettii*, *K. humilis*, and *K. unispora, Candida* represented by *C. boidinii* and *C. parapsilosis* species, and *Saccharomyces* represented by *S. cerevisiae*. Note that *C. parapsilosis* has recently been classified as biosafety level 2 (BSL-2) microorganism (JORF, 2021).

### 3.6 Microbial community diversity determined by metabarcoding analysis

#### 3.6.1 Mock community

A total of 42,395 high-quality reads of 16S rRNA V5-V7 regions were obtained after pre-processing and filtering. Except for *L. mesenteroides* CIRM-BIA2378, which was not detected, the nine other species of the mock were retrieved. However, *A. persici* was the only one identified at the species level. *E. cloacae* was identified at the family level only and the seven other species at the genus level. For three species, we obtained multiple different ASVs assigned to the correct species and multiple ASVs assigned to the correct genus: *L. plantarum* (2 ASVs at the species level/22 ASVs at the genus level), *P. pentosaceus* (1 ASV/2 ASVs), and *L. lactis* (1 ASV/28 ASVs). The respective abundance of each, which theoretically represented 10% of the mock, varied from 49% for *L. plantarum* to 0.2% for *H. casei*, while the percentages for *E. cloacae*, *L. lactis*, *E. faecium, G. cerinus*, *L. brevis, A. persici*, and *P. pentosaceus* were 16, 15, 11, 3, 2, 2, and 0.3%, respectively.

#### 3.6.2 DNA extraction results and metabarcoding results

DNA extraction led to sufficient amounts for 71 of the 75 collected samples, while the last four samples were discarded (S046, S211, S222, and S224). DNA concentration varied from 1 to 53 ng/μL with an average of 15 ng/μL. The average ratio A260/A280 was 1.73 ± 0.63 ng/μL. Only 27 samples had a DNA concentration superior to 10 ng/μL, the theoretical minimal concentration required by the sequencing platform, but all were, however, sent to be sequenced.

For the control, the total read numbers of the “kitome” samples performed in duplicate were 257 and 202, respectively, corresponding to *Enterobacteriaceae* (67%) and *Bacillaceae* (23%).

The 71 duplicate sample sequencing data were extremely similar. Duplicates showed a similar number of reads and a similar ASV profile (data not shown). The average standard deviation of the number of total reads was 7,966 (i.e., 0.09% of the total reads). Among the 71 samples, 24 had very few reads (≤5,000) and were thus discarded. All but one corresponded to aged samples (from 120 to 1540 days of fermentation), two of these samples being the oldest ones (over 4 years old). The 47 samples retained for metabarcoding were younger, and more DNA was recovered (median values of 75 days and 11.5 ng/μL versus 255 days and 1.8 ng/μL for the 28 discarded samples). We, therefore, kept 47 samples (Supplementary Table S1) for further analysis, with all of them having >16,000 reads (average number of reads per sample 116,988 ± 36,897).

For the 47 samples, a total of 5,498,414 high-quality reads of 16S rRNA V5-V7 regions were obtained. They formed 398 ASVs, divided between *Firmicutes* (199 ASVs, 81% of total reads) and *Proteobacteria* (199 ASVs, 19% of total reads), with a total of 5 orders, 14 families, and 37 genera. ASVs were identified at least at the family (resp. genus and species) level for 93% (resp. 71 and 10%) of them. Almost all multi-affiliated taxa, with ambiguous taxonomy, were members of the *Enterobacterales* order. The average number of ASVs per sample was 46 ± 19.

The number of total and valid reads and the alpha diversity indexes are given in Supplementary Table S5. Valid reads represented 78% of total reads, on average. Alpha diversity indexes showed large variations depending on the sample. The observed richness ranged from 14 to 101, with an average value of 44, whereas the Shannon index ranged from 0.16 to 2.96, with an average value of 1.65 (Supplementary Table S5). Alpha diversity indexes did not significantly differ according to the category of vegetable (root/bulb, fruit, leaf/stem, or mix, *p* = 0.61 for richness and *p* = 0.45 for Shannon, ANOVA) (Supplementary Figure S4).

Beta diversity analyses were performed on Jaccard distances to assess the structuring effect of several variables: vegetable type, category, sample age, LAB, and yeast viable counts. The only significant effect was age (*p* < 0.001, PERMANOVA), but it explained a small fraction of the observed beta dispersion (*R*^2^ = 4.2%). Vegetable category and pH were borderline significant (*p* = 0.06, PERMANOVA).

A clustering resulting from metabarcoding ASV data identified four clusters (Supplementary Figure S5), differing mostly in alpha diversity and the abundance of some LAB ASV, e.g., ASV2: the main *Lactococcus* genus ASV, more abundant in clusters C1 and C4, and ASV3 and ASV4, the two main ASV for *Levilactobacillus* genus, more abundant in clusters C2 and C3 and in clusters C1 and C4, respectively.

**Figure 5:**
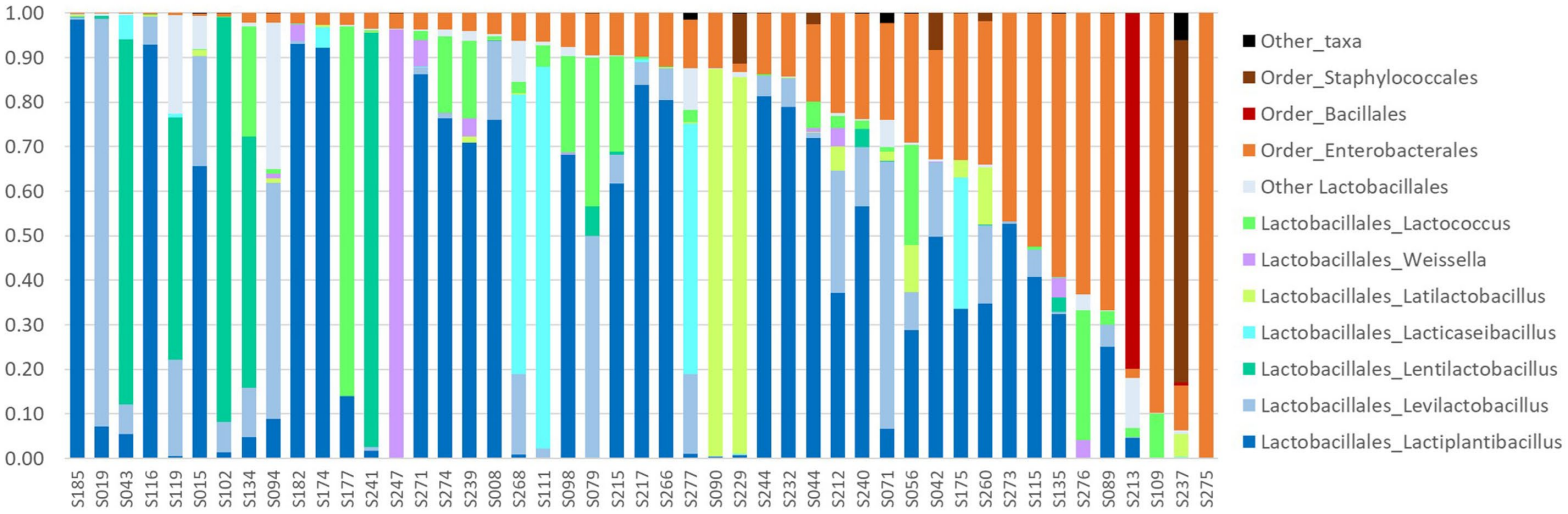
Relative abundances of LAB and some bacterial orders in the 47 homemade fermented vegetables successfully analyzed by metabarcoding targeting the 16S RNA gene. Samples are sorted by order of (i) decreasing proportion of *Lactobacillales* order, shown in different shades of blue, green, and purple according to the genus, and (ii) increasing proportion of *Enterobacterales* order.

At a taxonomic level, metabarcoding results showed that most samples had similar composition, with a bacterial community largely dominated by *Lactobacillales*, i.e., LAB, followed by *Enterobacterales* (Figure 5 and Supplementary Table S6).

*Lactobacillales* members (79% of total reads, 177 ASV) were present in all samples but one (S275) with a median abundance of 90% of the reads of the sample. They were mainly members of the *Lactobacillaceae* family (Supplementary Table S6). The four main genera identified were *Lactiplantibacillus*, followed by *Levilactobacillus*, *Lentilactobacillus*, and *Lacticaseibacillus*, and were present with abundance >5% in 70, 47, 13, and 13% of samples, respectively (Figure 5). A third of the 45 *Lactiplantibacillus* ASVs were identified at the species level, as *L. pentosus*, *L. fabifermentans*, *L. plantarum*, and *L. paraplantarum*. *Levilactobacillus* genus was represented by 36 ASVs, of which only 4 were identified at the species level (*L. yonginensis, L. brevis*, and *L. koreensis*). *Lentilactobacillus* genus was represented by 25 ASV with only one species identified, *L. farraginis*, while *Lacticaseibacillus* genus was represented by five ASV with only *L. paracasei* identified. Three other *Lactobacillales* members showed abundance over 1%: *Weissella, Secundilactobacillus*, and *Lactococcus* genera. The other genera, including *Leuconostoc* and *Pediococcus*, were detected at a very low abundance (less than 0.1% of total reads, Supplementary Table S6). Some samples differed from the others by the presence of a dominant but less common LAB taxon, i.e., *Latilactobacillus* was found dominant in two samples (S090 and S229), *Lacticaseibacillus* dominant in three samples (S111, S268, and S277)*, Lactococcus* dominant in sample S177, and *Weissella* dominated S247 profile (Figure 5).

The second main bacterial group, *Enterobacterales* (19% of total reads, 191 ASV), were present at an abundance above 1% in 91% of samples. They were mainly represented by two families *Enterobacteriaceae* and *Yersiniaceae* (Supplementary Table S5). *Enterobacterales* were significantly (value of *p* = 0.01007) most frequently observed at a higher abundance (> 10%) in the youngest samples (<200 days) than in the oldest ones (*p* < 0.02).

Two samples differed from the others by the dominance of *Bacillales* and *Staphylococcales*, respectively. *Bacillales*, represented by *Bacillus* and *Oceanobacillus*, dominated in sample S213, and *Staphylococcales*, represented by *Staphylococcus*, in sample S237 (Figure 5).

### 3.7 Multivariate analysis of the samples with metabarcoding results

**Figure 6:**
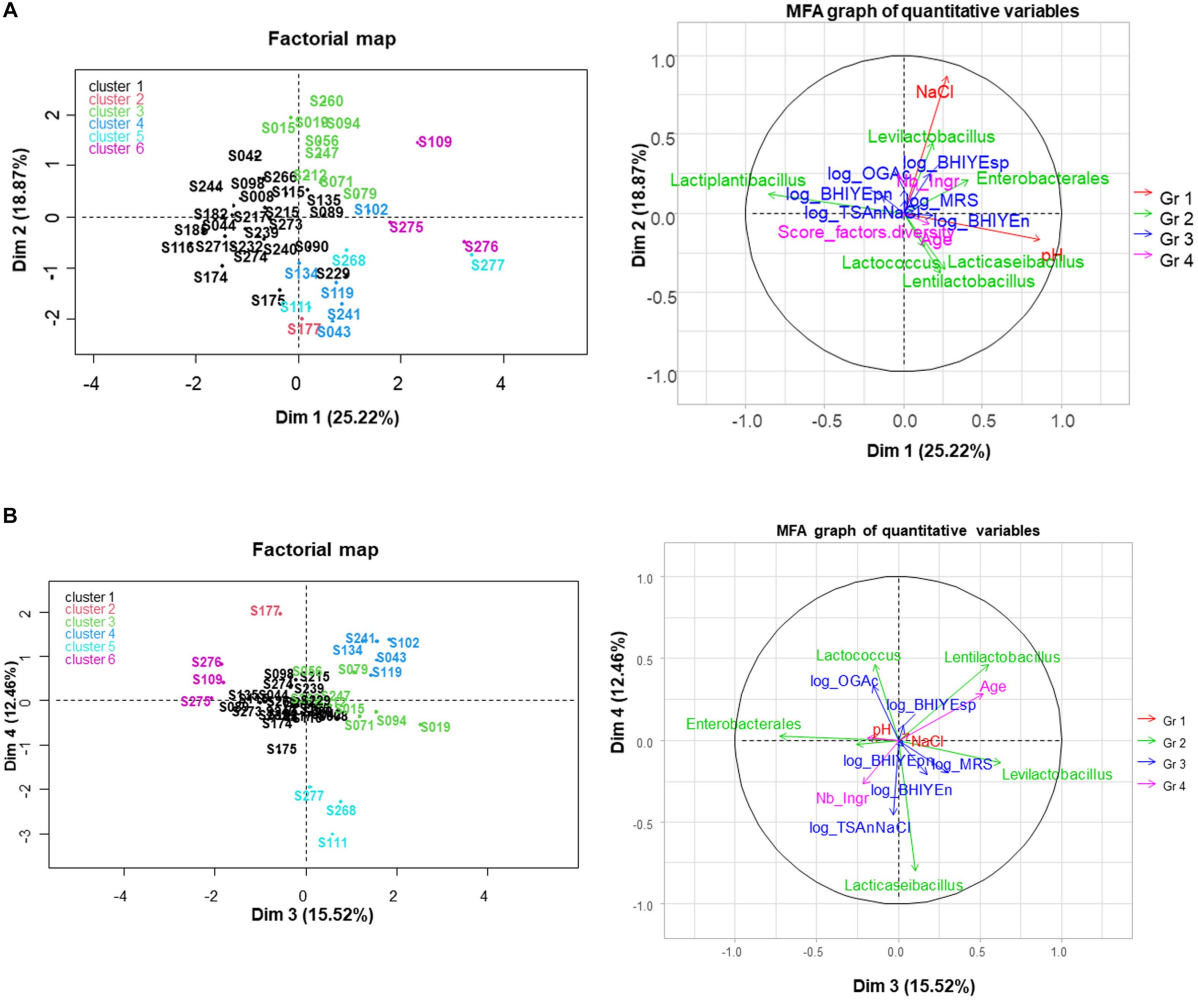
Multiple factor analysis (MFA) performed for 45 samples with metabarcoding results. The variables included as active variables were compositional variables (pH and NaCl content, depicted in red) and the six main taxa from metabarcoding analysis (depicted in green); quantitative variables were used as supplementary variable: descriptors of samples (age, number of ingredients, “diversity score,” depicted in pink) and plate counting results on six media, depicted in blue. Samples are colored according to a hierarchical clustering analysis made from MFA data. **(A)** Dim 1-Dim 2 plots; **(B)** Dim 3-Dim 4 plots.

A multiple factor analysis was performed on samples analyzed by metabarcoding to investigate the links between metabarcoding data and the microbial and biochemical composition of samples and some other descriptors (Figure 6A). Two atypical samples, S213 and S237, which contained *Staphylococcus* and *Bacillus* as main ASV taxon, respectively, were used as supplementary samples only for this analysis. Compositional variables (pH and NaCl) and the six main taxa from metabarcoding analysis (abundance >3%) were used as active variables. The other variables were used as supplementary variables, either quantitative: descriptors of samples (age, number of ingredients, “diversity score”) and plate counting results on six media, or qualitative variables: type and category of vegetables, type of cutting, presence/absence of viable bacteria on the last five enumeration media, and clusters from ASV shown in Supplementary Figure S5.

The first axis, which explained 25.2% of total variability, was positively correlated with high pH and negatively correlated with the proportion of *Lactiplantibacillus* observed in the metabarcoding analysis. The second axis (18.9% variability) was mainly correlated with the NaCl content. The five other metabarcoding-derived taxa were better described on the third or fourth dimensions of MFA, which explained 15.5 and 12.5% of total variability, respectively (Figure 6B). All the other quantitative variables appeared as poorly represented.

Regarding individuals, six clusters were distinguished from a hierarchical clustering analysis made from MFA data (Supplementary Table S7). The first one, represented in black, was the largest and contained samples with a high fraction of *Lactiplantibacillus* taxon (v-test = +5.0), a low fraction of *Levilactobacillus* (v-test = −2.8) and *Lentilactobacillus* (v-test = −2.4), and a low pH (v-test = −3.0) and was stored a relatively short time (mean age 108 days, v-test = −2.4). The second cluster contained only sample S177, shown in red, which contained a high abundance of *Lactococcus* (v-test = +5.2) and low viable LAB concentrations. Cluster 3, shown in green, contained nine samples that were associated with *Levilactobacillus* taxon and a high salt content (mean value 16.5 g/L, v-test = +3.9); it was also significantly associated with high concentrations of spore-forming bacteria (v-test = +2.4) and thinly cut samples (v-test = +2.7). Cluster 4, shown in blue, contained five samples with *Lentilactobacillus* as the main taxon. It was also associated with high age (531 days on average) (v-test = +3.9) and with beetroot (3 of the 5 beetroot samples, v-test = +2.7). Cluster 5 contained three samples, shown in light blue, which contained a high fraction of *Lacticaseibacillus* (v-test = +6.3). Cluster 6, shown in pink, contained three samples associated with the *Enterobacterales* taxon and a high pH (average pH value of 4.5, v-test = +3.6). It was also associated with the presence of alive bile-resistant *Enterobacteriaceae* (2 of the 4 VRBG-positive samples, v-test = +2.4) and rough cutting (v-test = +2.0). Note that the clustering resulting from the MFA markedly differed from the one obtained from metabarcoding ASV data shown in Supplementary Figure S5 and the qualitative variable ‘cluster from ASV’ was not associated with any of the clusters from MFA.

## 4 Discussion

### 4.1 A wide variety of homemade fermented vegetable samples collected

The manufacture of homemade fermented vegetables appears to increase in Western countries, likely encouraged by mainstream publications and social media, which popularize do-it-yourself approaches and promote fermented products. In contrast, in many Eastern European and Asian countries, fermented vegetables have been part of the tradition (Sõukand et al., 2015; Tamang et al., 2020). For example, a wide variety of plants (leaves, roots, bulbs, stalks, shoots, fruits, or seeds from 47 botanical taxa) have been until recently or are currently lactofermented, as a main or additional ingredient (Sõukand et al., 2015; Zhang et al., 2021).

Our objective was to characterize the practices and the microbiota of homemade fermented vegetables. Crowdsourcing thus offered a relevant strategy to collect a large panel of samples from citizens. It was done in the frame of the project FLEGME, a citizen science project that gathers citizens, SMEs, agricultural schools, culinary journalists, and researchers. Citizen sciences enable the collection of samples and/or data from large non-professional groups (Minet et al., 2017). Crowdsourcing initiatives are more and more used in biological sciences to collect environmental observations and/or biological samples, with the American gut project as an emblematic example (McDonald et al., 2018).

This study is the first, to the best of our knowledge, which characterized such a variety of homemade fermented vegetables. The other published reports targeted only one or a few types of products, such as sauerkraut (Liu et al., 2021) or carrot juice (Wuyts et al., 2018). To favor the variety of fermented vegetables collected, the citizens involved were let choose the samples sent among the ones they produce for their personal consumption, without directing their collection so as to get, for example, similar numbers of each type of vegetable. The recipes and manufacturing practices used to prepare these samples, described by the citizens, showed that the fermented vegetables collected were manufactured under very similar conditions regarding salting and temperatures of fermentation and storage. In contrast, they exhibited a wide variety in terms of (i) composition, i.e., number, nature, and mixture of vegetables, use of minor ingredients, herbs, and spices, (ii) degree of slicing: from entire small vegetables to finely grated or minced ones, and (iii) age at the date of collect. This extreme variety could explain, at least partly, the absence of clear data structure observed through both the targeted and untargeted statistical approaches used.

No impact of the type or the category of vegetables (root/bulb, leaf/stem, fruit), of the practices susceptible to increase diversity, e.g., the number of ingredients and the salt content, could be observed on the microbial composition of fermented vegetables. Similarly, in a study on 78 kimchi samples, no obvious trend with respect to the major ingredient was observed (Lee et al., 2017). In contrast, in a study designed to track the sources of kimchi microbes, some raw ingredients were identified as main microbial sources, and their role was highlighted in microbial community assembly (Song et al., 2020).

With regard to salt, its prominent role in the fermentation of vegetables has been reported. For example, salt concentration ranging from 1.72 to 4.42% was the major factor determining bacterial composition, rather than geographical region or major ingredient, in a large set of commercial and household kimchi samples (Lee et al., 2017). In the present study, only three samples had a concentration > 3.5% (Figure 2), and two highly salted samples analyzed by metabarcoding contained large (> 78%) proportions of *Bacillales* or *Staphylococcales* (Figure 5). The other samples collected had a fairly narrow range of NaCl content (96% of samples comprised between 0.4 and 2.2%, median value of 1.0%), compared to the results observed in similar studies on Asian fermented vegetables: 1.72 to 4.42% in Korean kimchi (Lee et al., 2017); 0.16 to 8.2% in Chinese homemade sauerkrauts (Liu et al., 2021). The larger range of salt concentration observed in Asian products may be due to the fact that recommendations cover a broader range of NaCl concentrations, e.g., the use of 0.5 to 3.5% brine (Yang et al., 2020). Lastly, it is important to highlight the huge discrepancies between the salt content declared in the recipe and the one actually measured. The use of dry salting for small amounts of vegetables could be involved in these discrepancies since it is difficult to accurately weigh small salt amounts at the kitchen scale. Accordingly, the eight samples with NaCl >2% were all dry-salted, and the proportion of high-salted (> 1.8% NaCl) samples was greater among dry-salted samples than among brined samples (value of *p* = 0.067).

Another factor that may explain the absence of data structure is the intrinsic diversity of the microbial community in spontaneously fermented products (Tamang et al., 2016; Gänzle, 2022). LAB are widespread in nature but represent less than 1% of the total bacterial population of raw vegetables, whose microbiota is generally dominated by aerobic bacteria such as pseudomonads, enterobacteria, and yeasts (Buckenhueskes, 2015). Data on LAB concentrations in raw vegetables show a huge variability, ranging from <1 to over 8.5 logCFU/g on aerial surfaces and from 2 to 4 logCFU/g on root vegetables (Yu et al., 2020).

### 4.2 Lactic acid bacteria dominated the microbial community of most samples

Both the culturomics and metabarcoding approaches that we combined to investigate the microbial community of fermented vegetables showed that LAB were the dominant bacterial group.

For metabarcoding, the results showed that LAB, i.e., members of the *Lactobacillales* order, were the dominant taxon, with a median abundance value of 90%. This highlights their ubiquity in varied fermented vegetables, as widely reported in sauerkraut, kimchi, and some other fermented vegetables, in studies that used culture-dependent and/or independent approaches (Thierry et al., 2023).

For culturomics, 15 media/conditions of culture were used to target varied microbial groups, considered as beneficial, undesirable, or pathogenic. The highest viable concentrations were observed on MRS, the medium used to enumerate LAB, on which nearly all the clones isolated were effectively identified as LAB. LAB may also have been the main bacteria that grew on some of the other culture media used, as on BHI-YEn, a rich medium used to enumerate total aerotolerant bacteria, as suggested by the high correlation between concentrations on these two media. In agreement, in another study using the latter medium and TSAn-NaCl, used to target halotolerant bacteria, most of the isolates identified from 1-month-old fermented carrot and cabbage were LAB (Valence, personal communication). Similarly, in paocai, a Chinese traditional fermented cabbage, LAB concentrations exceeded the bacterial concentrations enumerated on a rich medium at the end of fermentation (Wang et al., 2020). In our study, the number of LAB viable concentrations only depended, in a non-linear way, on the age of products, and no impact of manufacturing practices was observed. The considerable variations of the viable LAB numbers in retail fermented vegetables, which range from 10^1^ to 10^8^ viable LAB/g based on geographical region and sampling time, were recently underlined in a review on fermented foods as a dietary source of live organisms (Rezac et al., 2018).

In contrast with culturomics results, metabarcoding also showed that *Enterobacterales* members were the second dominant taxon, present at a median abundance of 9%. *Enterobacterales*, mainly represented by members of the *Enterobacteriaceae* family, are commonly found via metagenomic approaches in fermented vegetables (Thierry et al., 2023). They are associated with many raw materials and are common in the initial phases of various spontaneous food fermentations including vegetables (Kłapeć et al., 2016; Mudoor Sooresh et al., 2023).

The respective abundance of LAB and *Enterobacteriaceae* varies through the literature. For example, the abundance of *Enterobacteriaceae* members was about 50% in 1-month-old sauerkraut (Müller et al., 2018), about 25–50% in 6-month-old cucumbers (Świder et al., 2021), about 25% in 1-month-old fermented carrot juice, mainly represented by *Klebsiella* and *Citrobacter* (Wuyts et al., 2018), and about 5% in 12-day fermented radish (Liu et al., 2020). The quite large LAB and low *Enterobacteriaceae* abundances that we observed in the present study could be partly due to the high age of the samples. DNA was actually extracted from pelleted bacteria, thus excluding the DNA potentially released from lysed, non-pelleted, cells. Some bacteria may have lysed during long periods under the acidic conditions of fermented vegetables. A possible greater lysis of *Enterobacteriaceae* compared to LAB would have thus induced an underestimation of the former compared to the latter, at least in the more aged samples, but this point is poorly documented (Nielsen et al., 2007). The negative relationship observed in the present study between the abundance of *Enterobacterales* and the age of the samples supports this hypothesis.

LAB are known to outcompete *Enterobacteriaceae* during fermentation, as shown by studies that characterized dynamic changes, e.g., in fermented carrot juice (Wuyts et al., 2018). Our results illustrate the importance of culture-independent approaches to get an idea of the whole microbial community of fermented products when kinetic monitoring is not feasible.

### 4.3 A few taxa dominate the lactic acid bacteria population

Two genera, *Lactiplantibacillus* and *Levilactobacillus*, appeared as the main LAB taxa, as consistently shown by both culturomics and metabarcoding approaches. Culture-dependent results showed 11 LAB genera within the 93 isolates identified, with half of the isolates identified that belonged to *Lactiplantibacillus* or *Levilactobacillus* (26 and 25% of LAB isolates, respectively). In agreement, the ASV related to these two genera identified by metabarcoding represented 52 and 15%, respectively, of the total reads within the *Lactobacillales* order. These results agree with the literature data considering the role of both these species. For example, the four main species listed for 18 traditional fermented vegetables were *L. plantarum* and *L. brevis*, followed by *P. pentosaceus* and *L. mesenteroides* (Montet et al., 2014), and the most frequently isolated LAB species isolated from 30 traditional fermented vegetables were the same four species (Thierry et al., 2023)*. L. plantarum*, *L. brevis*, and *Pediococcus ethanolidurans* were also cited as the most frequently LAB species isolated from homemade Chinese sauerkraut (Liu et al., 2021) and *L. plantarum, L. brevis*, and *L. mesenteroides* from spontaneously fermented leek, in addition to *Latilactobacillus sakei* and *Levilactobacillus parabrevis* (Wouters et al., 2013b). In agreement, the main bacterial genera identified in fermented vegetables through 16S-amplicon-based metagenomic analyses over the last decade are the former *Lactobacillus* genus, followed, to a lesser extent, by *Weissella*, *Leuconostoc*, *Pediococcus*, and *Lactococcus* (Thierry et al., 2023).

The main differences between the present results and literature data are the absence of *Leuconostoc* and, to a lesser extent, the low abundance of *Pediococcus* in metabarcoding data, and the low amounts of isolates of *Weissella* and *Lactococcus*. Two possible and combined factors may explain these differences: some specific characteristics of the samples collected and some bias in metabarcoding analysis.

Regarding *Leuconostoc*, only a few isolates of this genus were identified from those isolated from MRS medium, on the one hand (Figure 4), and this genus was nearly undetected by metabarcoding (abundance 0.04%), markedly lower than expected, on the other hand. Moreover, the abundance of multi-affiliated *Lactobacillaceae* ASV accounted for only 0.34% and thus cannot explain the absence of *Leuconostoc*. The low number of *Leuconostoc* isolates collected is likely due to the high age of the samples collected (median value of 180 days, >30 days for all samples except five). *Leuconostoc* sp., in particular *L. mesenteroides*, is known as the major species involved in the early stage of vegetable fermentation, for example, in sauerkraut (Pederson and Albury, 1969) and in carrot juice (Wuyts et al., 2018). It is thus likely that the leuconostoc that initially grew had died in most samples at the stage of collect. Concerning metabarcoding results, the absence of detection of *Leuconostoc* in the mock community, which contained an *L. mesenteroides* strain at 10%, showed that this taxon was not amplified and thus explains the very low abundance of *Leuconostoc* taxon detected in fermented vegetables. These results can be explained by the primer pair used, 799F-1193R, whose sequence presented two mismatches with 16S rRNA *Leuconostoc* sequence, thus preventing their amplification. These primers, which target the hypervariable V5-V7 region of 16S rRNA, were chosen (i) among the primers commonly used to analyze plant-associated bacterial communities, to limit affinity for non-targeted DNA such as plastid (mostly chloroplast) and mitochondrial DNA, (ii) to amplify rhizosphere bacteria potentially present in vegetables, according to Beckers et al. (2016), and (iii) for their reported performance among seven 16S rRNA regions, on four mock communities tested (Barb et al., 2016). As expected, the sequences obtained in the present study were not contaminated by chloroplastic and mitochondrial DNA, confirming their efficiency in this aspect. These results underline the delicate balance in the choice of relevant primers, by at the same time considering the universality of primers, at least within the expected community studied, and avoiding the background noise due to the raw materials analyzed. Read numbers could also partly depend on the 16S copy number, which varies according to the species (Cocolin and Ercolini, 2008; Stoddard et al., 2015). However, *L. plantarum* harbors five copies of the 16S rRNA gene, such as *L. brevis*, *L. lactis*, and *L. pentosaceus*, so the number of 16S copies cannot explain *L. plantarum* overestimation in the mock community used in the present study. The DNA extraction yield, amplification efficiency, and sequencing fidelity are other biases that could explain that each species of the mock did not show the same abundance. The results of mock community analysis strongly underline that metabarcoding should anyway be considered as semi-quantitative only. For *Pediococcus*, similarly, the underestimation of its abundance in the mock community can explain the low abundance of this taxon in the metabarcoding results of the present study.

*Lactococcus* taxon was largely detected by metabarcoding even at sub-dominant levels, while only one isolate was identified. This apparent discrepancy could result from a decrease in viability of these bacteria, well known for their susceptibility to cell lysis (Boutrou, 1996), during the long storage of many of the samples studied here and/or the fact that they hardly grew on MRS. Regarding *Weissella* taxon, it was dominant only in a 2-month-old parsnip sample (S247), while its dominance has been frequently reported, e.g., in fermented radish juice (Xu et al., 2018) and other vegetables (Thierry et al., 2023). The rather low numbers of *Weissella* strains isolated compared to literature data could be also due to the potential decrease in viability during storage. A few other LAB taxa, i.e., *Lacticaseibacillus* and *Latilactobacillus*, were dominant in only one to three samples (Figure 5). The sole isolate identified of *Secundilactobacillus* was isolated from the sample in which this taxon showed the highest abundance (S094, over 30%).

### 4.4 Yeasts are common members of the fermented vegetable microbial community

Yeasts were detected in nearly half of the samples, at a median count of 4.4 log CFU/g. The presence of yeasts in diverse fermented vegetables has been occasionally reported, at concentrations ranging from 0.3 to 4.6 logCFU/g (Nout and Rombouts, 2000), e.g., in Chinese sauerkraut yeasts reached an average count of 3.7 logCFU (Liu et al., 2021) and 4 logCFU in Chinese *paocai* (Wang et al., 2022a).

A large number of yeast genera have been reported in fermented vegetables. For example, the identification of yeasts isolated from Chinese homemade sauerkraut samples showed *Candida*, *Saccharomyces*, *Pichia*, *Kazachstania*, *Issatchenkia*, *Torulopsis*, and *Zygosaccharomyces* as main genera (Liu et al., 2021). A metagenomic approach to a fungal community of a cabbage-based Chinese *paocai* showed 17 fungal taxa, among which *Candida*, *Kazachstania*, and *Debaryomyces* dominated (Wang et al., 2022b). Accordingly, our results, based on isolate identification only, highlighted a wide yeast diversity, with *Kazachstania* and *Saccharomyces* as prevalent genera, in line with the literature data.

Yeast growth was reported as more prevalent at high salt concentrations and can be associated with a pink defect in sauerkraut (Pederson and Albury, 1969), in agreement with our observation that the samples that contained yeasts were generally among the most salted ones. The role of yeasts in the fermentation of vegetables has to be further investigated. They are generally considered as spoilage microorganisms (Ballester et al., 2022) but can also be considered as contributors to the formation of flavor, as in Chinese *paocai*, in which *Debaryomyces hansenii* and *Kazachstania exigua* were shown to produce esters and alcohols, belonging to *paocai* key odorants (Wang et al., 2022b).

### 4.5 Other microbial groups and potential safety concerns

Homemade fermented vegetables are consumed without any microbiological or even pH control. In general, they are considered as edible if they have a typical acidic odor, as said in books for general readerships and blogs (Katz, 2012; Frédéric, 2014, 2021) The very low pH (median value at 3.6) observed in most of the collected samples made them belong to the FDA category of ‘acidified foods’, i.e., with a pH below 4.6, a value widely accepted as food safety threshold (FAO, 2007; Food and Drug Administration, 2023). It can be underlined here that the same pH can correspond to different total acid concentrations, depending on the buffer content of the juice or brine, and also on the proportion of acetic and lactic acid, due to their difference of dissociation constant (pKa of 3.86 and 4.76 for lactic and acetic acids, respectively) (Pederson and Bagg, 1944). Threshold values of 4 for pH and 1% for titratable acidity specifications have been laid down for the protected geographical indication (PGI) “Sauerkraut of Alsace” (Regulation (EU) No 1151/2012, 2018). In addition to the low pH of the samples, the absence of detection of the four targeted pathogenic bacteria and the dominance of LAB, which produce lactic and acetic acids and other organic acids with antimicrobial properties (Theron and Lues, 2011), strongly support the safety of most of these homemade products.

In our study, since there is no specific standard for fermented vegetables, we targeted several pathogens and bacterial groups that can include pathogen members. Some safety concerns can be raised for a few samples. Coagulase-positive staphylococci were detected at concentrations >100 in one sample and low concentrations (<200) of *B. cereus* of group II and IV, which may include cytotoxic strains in three samples, and a black colony of anaerobic sulfite-reducing spore-forming bacteria was observed in another sample. The presence of *Bacillus* sp. has previously been reported (Xu et al., 2018). *Bacillus* includes toxicogenic species and has been identified as the main producers of biogenic amines in fermented mustard (Yu et al., 2022), while some *Bacillus* species, e.g., *B. marcorestinctum*, are considered as beneficial for fermentation (Zhong et al., 2022). In contrast, no *E. coli*, *Salmonella*, and *L. monocytogenes* were detected. Several outbreaks linked to kimchi consumption contaminated by *Listeria*, *E. coli*, or norovirus have, however, occurred, suggesting that safety cannot be absolutely guaranteed, for reasons referring to both the raw materials and the pathogen adaptability (Patra et al., 2016). In another study that investigated pathogens in 68 fermented vegetables sold at the Phnom Penh market, *E. coli* and *Cronobacter sakazakii* were detected in 10 and 1%, of samples, respectively, but the pH ranged from 3.6 to 6.5 (Chrun et al., 2017).

Regarding the presence of *Enterobacterales* members, it is impossible to evaluate the potential risks associated with their presence since most (> 82%) *Enterobacterales* members were unidentified even at the genus level. *Enterobacteriaceae* are commonly identified in fermented vegetables through 16S-amplicon-based metagenomic analyses (Thierry et al., 2023). The main genera identified here were *Rahnella* (3.7% of *Enterobacterales* reads) and *Enterobacter* (3.3% of *Enterobacterales* reads). *Enterobacter* spp. have environmental reservoirs in water and soil, and *Enterobacteriaceae* are common in the rhizosphere (Kłapeć et al., 2016). *Enterobacter* has even been proposed as an potentially important genus for the production of flavor compounds in homemade Chinese sauerkraut (Yang et al., 2020). Aerotolerant Gram-negative bacteria, counted on BHI-YEnp, a rich non-selective medium, were commonly detected in fermented vegetables collected in the present study. The colonies that grew on this medium were not identified, but in another study that used the same medium and conditions to enumerate bacteria from 1-month-old fermented cabbage, many *Enterobacterales* taxa were identified within the eight isolates collected (three *Hafnia alvei* isolates, two *Serratia*, one *Enterobacter*, one *Kluyvera*, and one *Pantoea*, Valence, unpublished data). *H. alvei* belongs to the microbiota of some soft cheeses and is known to be capable to grow at an acidic pH (Mounier et al., 2008). In contrast, only four fermented vegetables showed colonies on VRBG, a medium that is specifically formulated to enumerate bile-tolerant *Enterobacteriaceae* species in dairy products and foods (Corry et al., 2003). Environmental factors such as temperature, pH, and nutrient level affect the sensitivity of enterobacteria to acid, as do the presence of phosphate and sodium and the extent of aeration (Rowbury, 1995), which could explain that enterobacteria were detected alive in a few samples despite their acidic pH values.

The use of starters has been proposed to improve and standardize the quality of fermented vegetables (Torres et al., 2020). In a study on fermented carrot juice, for example, controlled fermentation was inoculated with isolates to test them as potential starters (Wuyts et al., 2018). The results showed that only strains belonging to the most prevalent LAB preserved the fermentation dynamics. The capacity of starters to outcompete the autochthonous microbial community and use specific carbohydrates of raw materials is crucial, and autochthonous starters have been recommended for this purpose (Di Cagno et al., 2013). Several other studies explore the use of autochthonous LAB strains as starters, e.g., in vegetable mixtures (Gardner et al., 2001) and leeks (Wouters et al., 2013a), stressing the importance to gain access to autochthonous strains. The 93 LAB strains and the 33 yeast strains isolated have been deposited in the Biological Resource CIRM-BIA (see footnote 1) and CIRM-Levures,^3^ respectively, where they are available for the research community.

### 4.6 Conclusion

This study highlights the very large variety of currently household fermented vegetables and the recipes implemented. It is the first study, to our knowledge, to characterize such a variety of fermented vegetables, including very old samples. The data collected along the samples within the frame of this citizen science project also provide knowledge elements on the manufacture of fermented vegetables at a domestic scale.

This study also confirms the complementarity of culturomics and metabarcoding approaches to characterize food ecosystems, especially when kinetic monitoring is not possible. Using only one or the other approach would have led to a fragmentary vision. With a view to securing the production of homemade and artisanal fermented vegetables, this study opens up avenues for exploring in more detail some factors which could influence the microbial composition, such as the degree of cutting of vegetables and the salt content. New open questions remain to be explored, such as the role of yeasts in particular in flavor development, and the time course of the establishment of both undesirable and desirable microbiota.

## Data availability statement

The sequence data for this study have been deposited in the European Nucleotide Archive (ENA) at EMBL-EBI under accession number PRJEB60847 (https://www.ebi.ac.uk/ena/browser/view/PRJEB60847).

## Author contributions

AT: Conceptualization, Data curation, Formal analysis, Funding acquisition, Resources, Supervision, Validation, Visualization, Writing – original draft, Writing – review & editing. M-NM: Investigation, Methodology, Supervision, Writing – original draft. VC: Investigation, Methodology, Resources, Writing – original draft. A-SB: Data curation, Investigation, Writing – original draft. OP: Investigation, Writing – original draft. CG: Investigation, Writing – original draft. OR: Formal analysis, Methodology, Supervision, Visualization, Writing – original draft. MM: Formal analysis, Software, Visualization, Writing – review & editing, Writing – original draft. LM: Resources, Writing – original draft. FV: Conceptualization, Funding acquisition, Project administration, Supervision, Visualization, Writing – review & editing.

## Footnotes

^1^ https://collection-cirmbia.fr/

^2^ https://shiny.migale.inrae.fr/app/affiliationexplorer

^3^ https://cirm-levures.bio-aware.com/page/Home
