# Supplementary material for: Microbial communities of a variety of 75 homemade fermented vegetables
Source: Front Microbiol. 2023 Dec 15;14:1323424. doi: 10.3389/fmicb.2023.1323424 (PMC10757351; doi:10.3389/fmicb.2023.1323424)
Supplement: Supplementary file 2 [file Presentation_1.PPTX]

## Slide 1
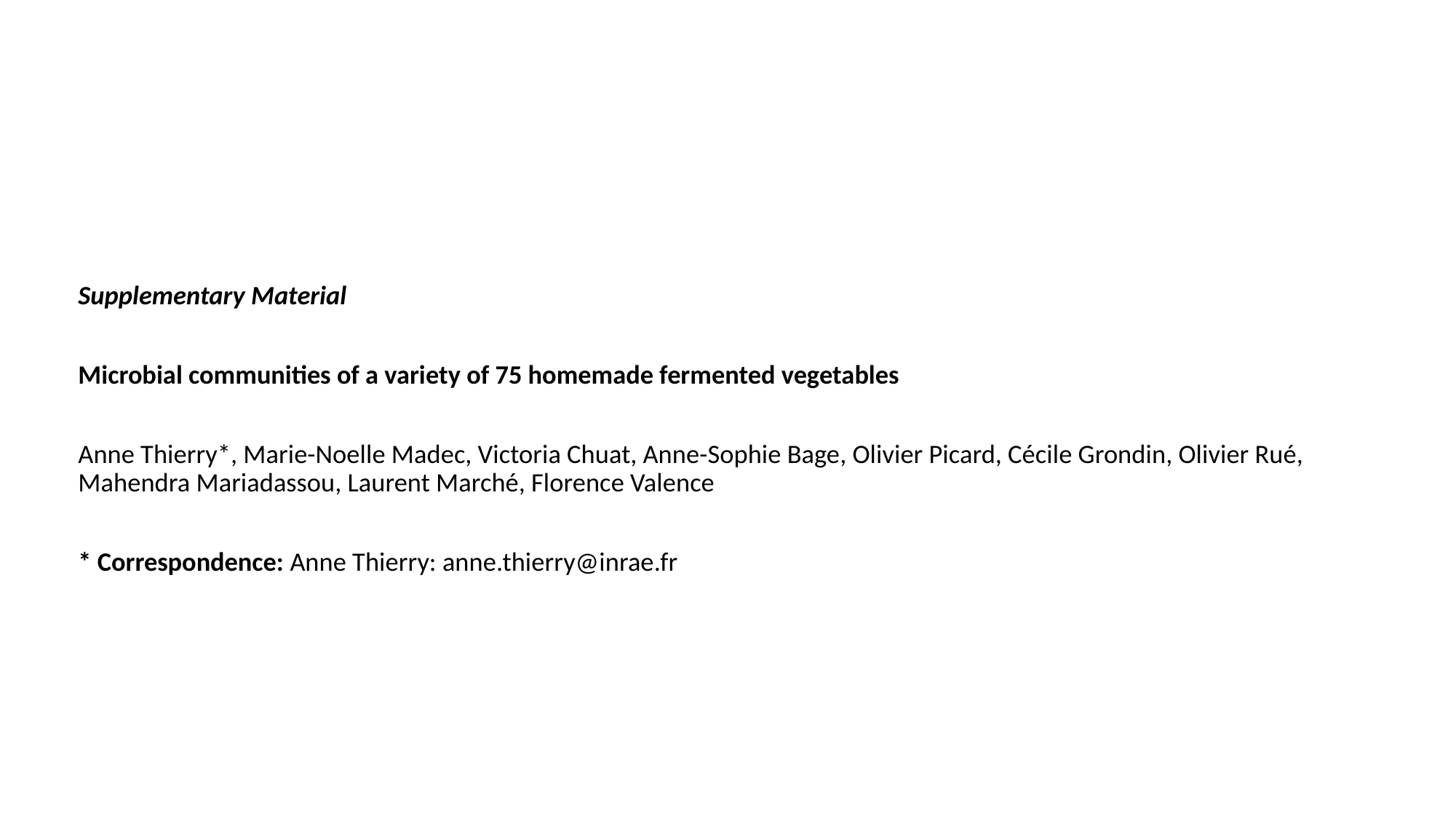

Supplementary Material
Microbial communities of a variety of 75 homemade fermented vegetables
Anne Thierry*, Marie-Noelle Madec, Victoria Chuat, Anne-Sophie Bage, Olivier Picard, Cécile Grondin, Olivier Rué, Mahendra Mariadassou, Laurent Marché, Florence Valence
* Correspondence: Anne Thierry: anne.thierry@inrae.fr

## Slide 2
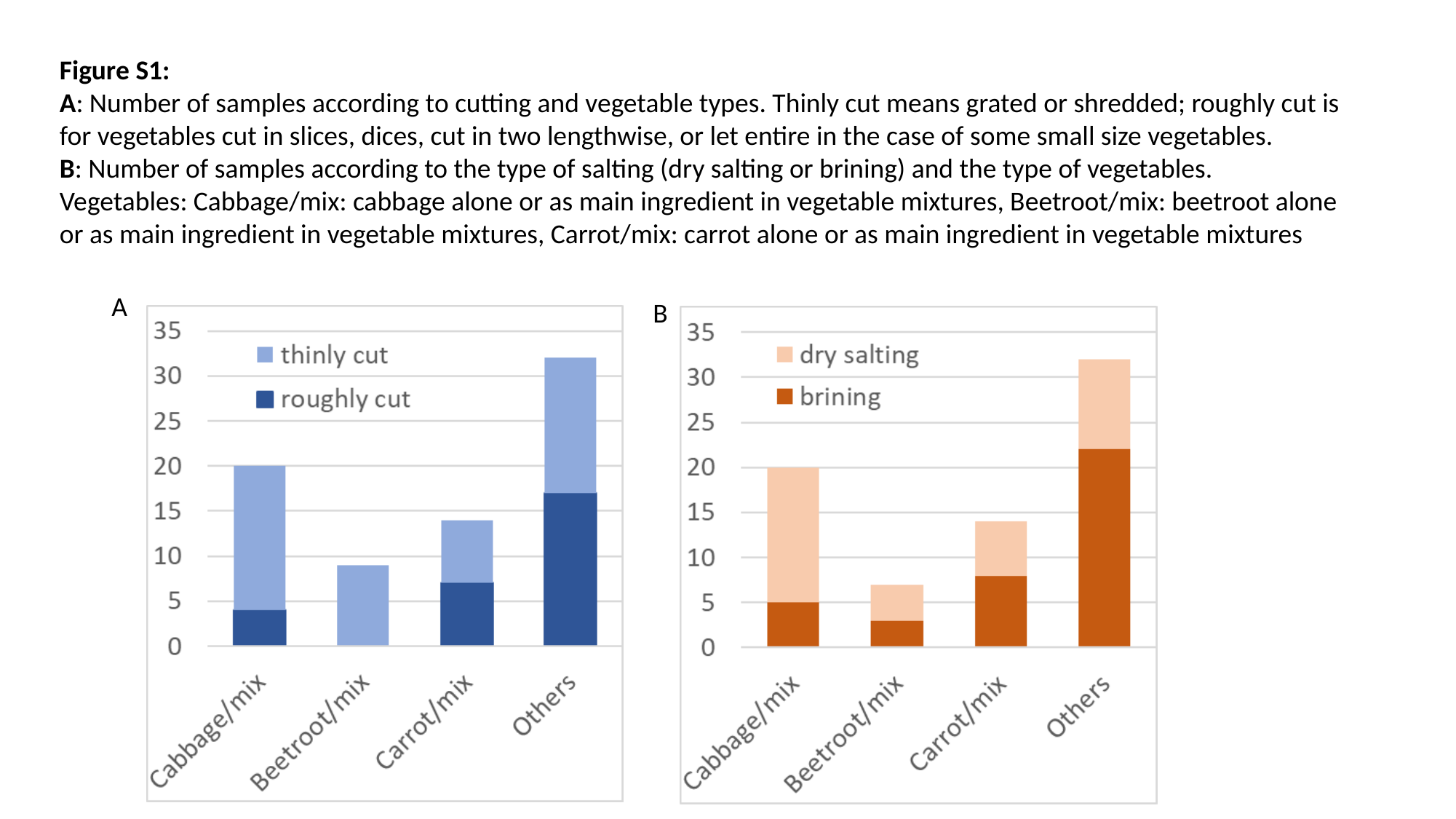

Figure S1:
A: Number of samples according to cutting and vegetable types. Thinly cut means grated or shredded; roughly cut is for vegetables cut in slices, dices, cut in two lengthwise, or let entire in the case of some small size vegetables.
B: Number of samples according to the type of salting (dry salting or brining) and the type of vegetables. Vegetables: Cabbage/mix: cabbage alone or as main ingredient in vegetable mixtures, Beetroot/mix: beetroot alone or as main ingredient in vegetable mixtures, Carrot/mix: carrot alone or as main ingredient in vegetable mixtures
A
B

## Slide 3
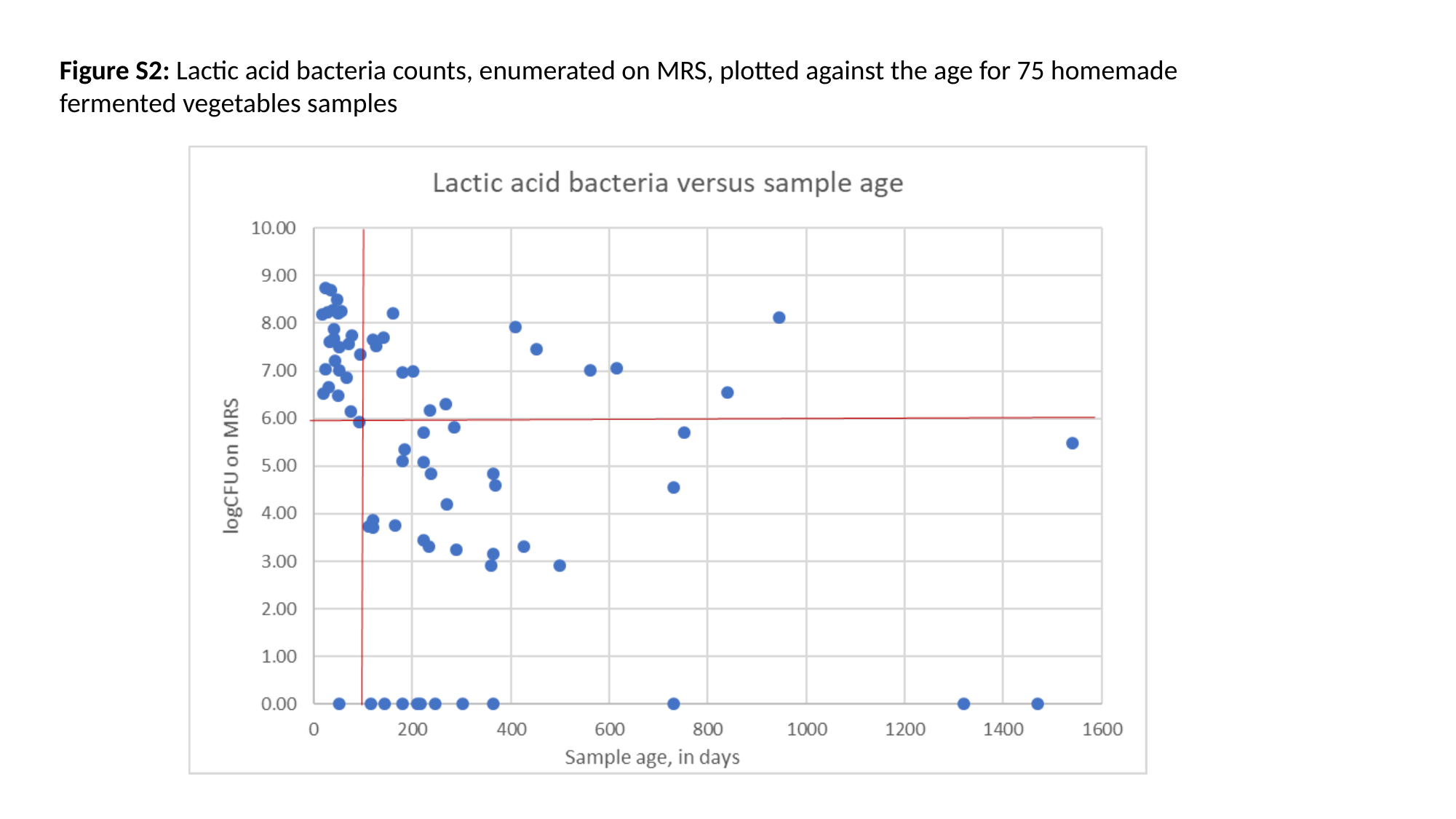

Figure S2: Lactic acid bacteria counts, enumerated on MRS, plotted against the age for 75 homemade fermented vegetables samples

## Slide 4
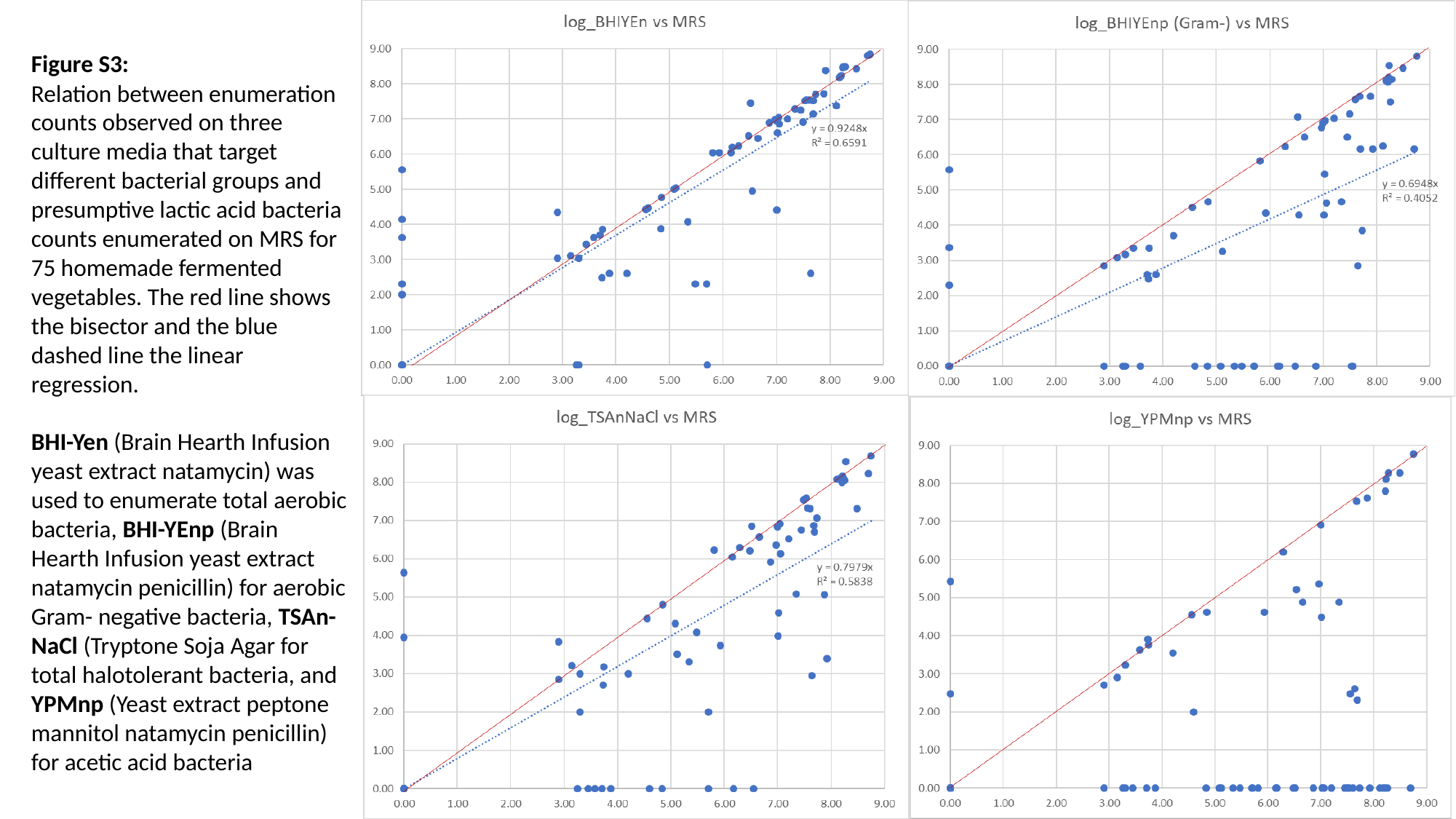

Figure S3:
Relation between enumeration counts observed on three culture media that target different bacterial groups and presumptive lactic acid bacteria counts enumerated on MRS for 75 homemade fermented vegetables. The red line shows the bisector and the blue dashed line the linear regression.
BHI-Yen (Brain Hearth Infusion yeast extract natamycin) was used to enumerate total aerobic bacteria, BHI-YEnp (Brain Hearth Infusion yeast extract natamycin penicillin) for aerobic Gram- negative bacteria, TSAn-NaCl (Tryptone Soja Agar for total halotolerant bacteria, and YPMnp (Yeast extract peptone mannitol natamycin penicillin) for acetic acid bacteria

## Slide 5
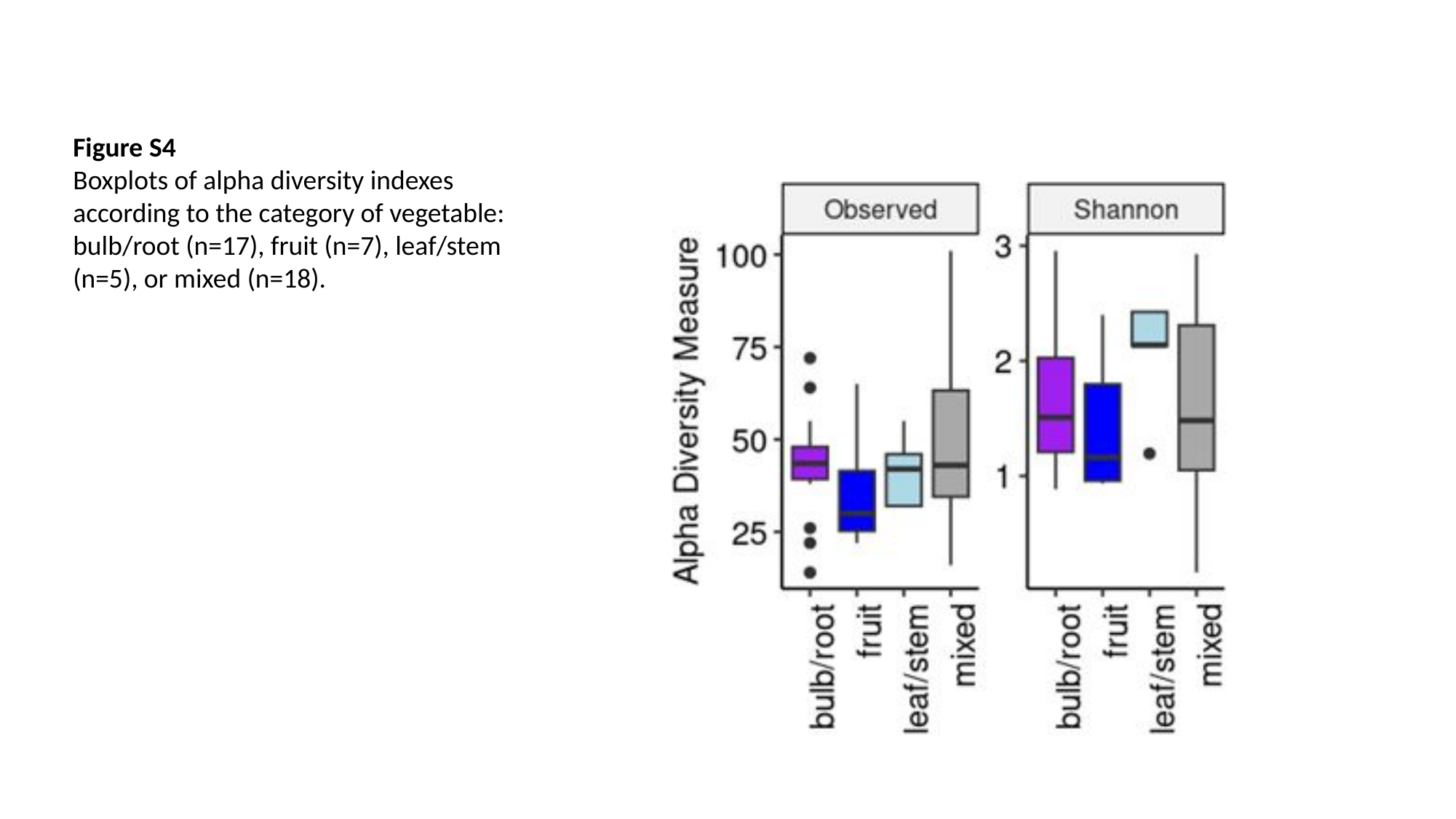

Figure S4
Boxplots of alpha diversity indexes according to the category of vegetable: bulb/root (n=17), fruit (n=7), leaf/stem (n=5), or mixed (n=18).

## Slide 6
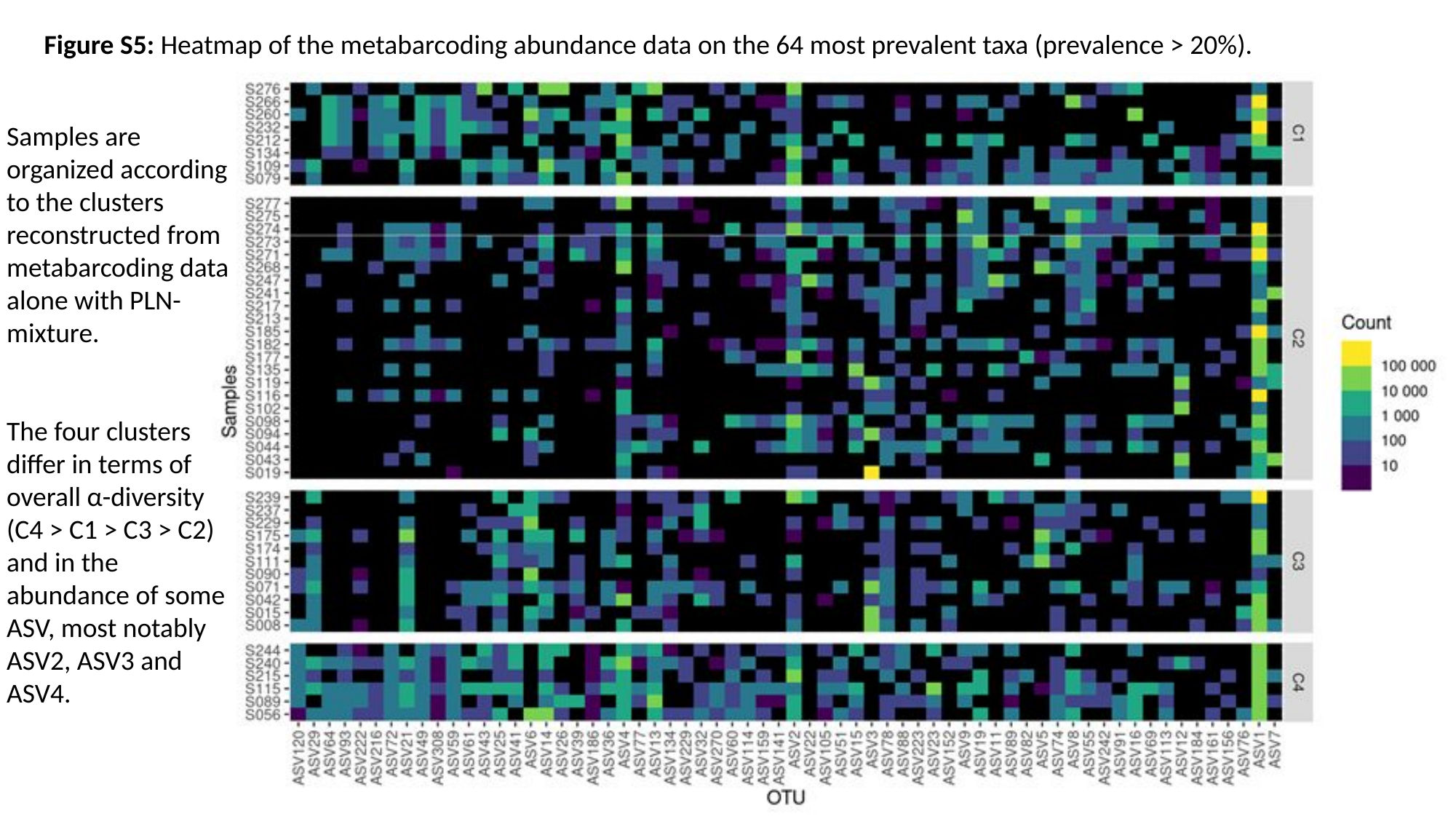

Figure S5: Heatmap of the metabarcoding abundance data on the 64 most prevalent taxa (prevalence > 20%).
Samples are organized according to the clusters reconstructed from metabarcoding data alone with PLN-mixture.
The four clusters differ in terms of overall α-diversity (C4 > C1 > C3 > C2) and in the abundance of some ASV, most notably ASV2, ASV3 and ASV4.
